# Scoping review of preclinical and clinical studies on the role of HMGB1 in heart disease

**DOI:** 10.1038/s44325-026-00119-4

**Published:** 2026-04-09

**Authors:** Shih-Hsuan Mao, Roger Guan-Jie Luo, Carl Lee, Jia-Ling Ruan, Lynn Williams, Alvaro Viñals Guitart, Kat Steiner, James K. K. Chan, Juan Enrique Berner, Andrew J. M. Lewis, Paul R. Riley, Jagdeep Nanchahal

**Affiliations:** 1 The Kennedy Institute of Rheumatology, Nuffield Department of Orthopaedics, Rheumatology and Musculoskeletal Sciences, University of Oxford, Oxford, UK; 2 Department of Plastic and Reconstructive Surgery, Chang Gung Memorial Hospital, Linkuo & Chang Gung University College of Medicine, Taoyuan, Taiwan; 3 Department of Oncology, University of Oxford, Oxford, UK; 4 Bodleian Health Care Libraries, University of Oxford, Oxford, UK; 5 Department of Plastic Surgery, Stoke Mandeville Hospital, Aylesbury, UK; 6 Division of Plastic and Reconstructive Surgery, Department of Surgery, University of Texas at San Antonio, San Antonio, TX USA; 7 Oxford Centre for Clinical Magnetic Resonance Research, University of Oxford, Oxford, UK; 8 Institute of Developmental and Regenerative Medicine, University of Oxford, Oxford, UK; 9 Department of Physiology, Anatomy and Genetics, University of Oxford, Oxford, UK

**Keywords:** Cardiology, Cell biology, Diseases

## Abstract

High Mobility Group Box 1 (HMGB1) is a critical regulator of cardiac injury and repair. However, there is conflicting evidence regarding whether HMGB1 is beneficial or deleterious. We evaluated and synthesised available evidence regarding the molecular functions as well as the clinical and therapeutic utility of HMGB1 in heart disease. We found that overall, the effects depend on the redox state and subcellular location, although most studies failed to identify these parameters clearly. Nuclear upregulation or exogenous administration of fully reduced HMGB1 was beneficial. The partially oxidised form of HMGB1 (dsHMGB1) in the cytoplasm depleted intranuclear HMGB1, and extracellular dsHMGB1 was proinflammatory. Clinically, elevated circulating HMGB1 levels correlate with disease severity and may be a useful prognostic biomarker. Future research should specify the subcellular location and redox state of HMGB1. The development of methods to identify the different redox isoforms would help uncover the therapeutic potential of this multifaceted protein.

## Introduction

Cardiovascular disease (CVD) is the leading cause of mortality worldwide, with ischaemic heart disease affecting ~200 million people annually^1^. A recent meta-analysis estimated that 3.8% of individuals aged under 60 and 9.5% of those over 60 years suffered myocardial infarction (MI) globally^2^. Despite advances in revascularisation strategies following MI, ~40% of patients develop heart failure (HF)^3,4^. An estimated 65 million adults worldwide suffer from HF^1,5^, and the prevalence continues to rise with an ageing population and increasing rates of obesity, diabetes, and hypertension. By 2030, the number of HF cases in the USA is projected to increase by 45%, reaching 8 million, with total direct medical costs surging to $53 billion^6,7^. Despite guideline-directed therapies, the 5-year survival rate for HF remains around 50%, underscoring the need for new treatments^5,8^.

A potential novel therapeutic avenue for improving outcomes in cardiac disease involves targeting alarmins, endogenous danger signals capable of modulating both pathological inflammation and physiological repair^9^. Among these, High Mobility Group Box 1 (HMGB1) is the most extensively characterised^9^.

HMGB1 is a highly conserved non-histone chromatin-binding protein whose function is dictated by its subcellular location and redox state^10^. In the nucleus, HMGB1 maintains chromatin structure and regulates DNA repair, replication, and transcription^10^. Under cellular or redox stress, nuclear HMGB1 translocates to the cytoplasm, primarily through modifications of nuclear localisation signals (NLS1, NLS2) via acetylation or deacetylation^11,12^. Other post-translational modifications, including ADP-ribosylation, phosphorylation, and methylation, also facilitate shuttling and intracellular signalling^12^.

HMGB1 can be actively secreted by immune cells, fibroblasts, cardiomyocytes, epithelial or endothelial cells^11–14^, or passively released from necrotic or damaged cells, when it functions as an alarmin^9,15^. The extracellular activity is determined by its redox state and corresponding receptor interactions^16,17^.

**Fig. 1:**
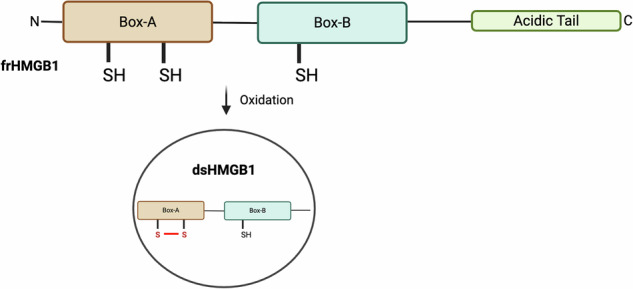
Schematic representation of HMGB1 structure. HMGB1 consists of two DNA-binding domains, Box A and Box B, followed by a C-terminal acidic tail. In the fully reduced form (frHMGB1), thiol groups are present at C23 and C45 in Box A, and at C106 in Box-B. Oxidation results in the formation of a disulfide bridge between the cysteines at C23 and C45, yielding disulfide HMGB1 (dsHMGB1). Created in BioRender. Mao, S-H. (2026) https://BioRender.com/ltw7v97.

HMGB1 consists of two structurally homologous DNA-binding domains (Box A and Box B) and a C-terminal acidic tail (Fig. 1). The three cysteine residues (C23, C45, C106) are critical to modulating it's function^16,18^. The fully reduced form (frHMGB1) promotes tissue repair and chemotaxis via CXCL12-CXCR4 signalling^19–23^. However, frHMGB1 can be partially oxidised into dsHMGB1, with a disulfide bridge between C23 and C45 in the Box A domain^24^. The resulting conformation change modulates receptor binding. dsHMGB1 interacts with Toll-like receptor-2 (TLR-2), TLR-4, and Receptor for Advanced Glycation End-products (RAGE) to promote inflammation^25^ and platelet activation^26^.

frHMGB1 has a half-life of ~17 min in human tissue fluids in vitro, converting entirely to dsHMGB1 within 50 min, whilst dsHMGB1 has a half-life 642 min^24^. In vivo, dsHMGB1 binds to haptoglobin, and following binding to CD163 on macrophages, the complex is cleared by endocytosis^27^. A study investigating HMGB1 dynamics in trauma patients identified two distinct peaks in the circulation^28^. The initial release occurred immediately following injury and declined rapidly, with a half-life of 26 min. Levels correlated with the extent of injury. This initial wave was likely due to the passive release of frHMGB1 from damaged tissue. The second peak emerged around 3 h and declined by around 6 h post-trauma, and was strongly predictive of deleterious outcomes and systemic inflammation. Although the redox states were not assessed, this secondary peak likely corresponded to the pro-inflammatory disulfide isoform (dsHMGB1)^28^. Fully oxidised HMGB1, when all cysteines are oxidised, lacks chemotactic or inflammatory properties^16^.

HMGB1 is elevated in serum and myocardial tissue in response to cardiac diseases^29–31^. Inhibition of HMGB1 has been shown to reduce inflammation and myocardial injury^29,32–39^ and exogenous administration can lead to worse outcomes^29,32–34,40–43^. However, HMGB1 inhibition can also be detrimental^30,44^, and exogenous administration or cardiac-specific overexpression may be beneficial in MI^31,45–47^ and HF^48–50^. These apparently contradictory effects have made it challenging to understand the role of HMGB1 in cardiac disorders.

The apparently contradictory effects of HMGB1 mean that in order to understand its potential as a therapeutic target, it is crucial to appreciate how its function in cardiac disease is modulated by its location (intracellular or extracellular), redox state, and specific disease context. Whilst previous reviews have highlighted conflicting data regarding these roles^16,17,51,52^, this scoping review with a systematic search provides a detailed update on the current understanding of HMGB1 in cardiac disorders, aiming to resolve these discrepancies by analysing and synthesising the available evidence.

## Results

### Overview

**Fig. 2:**
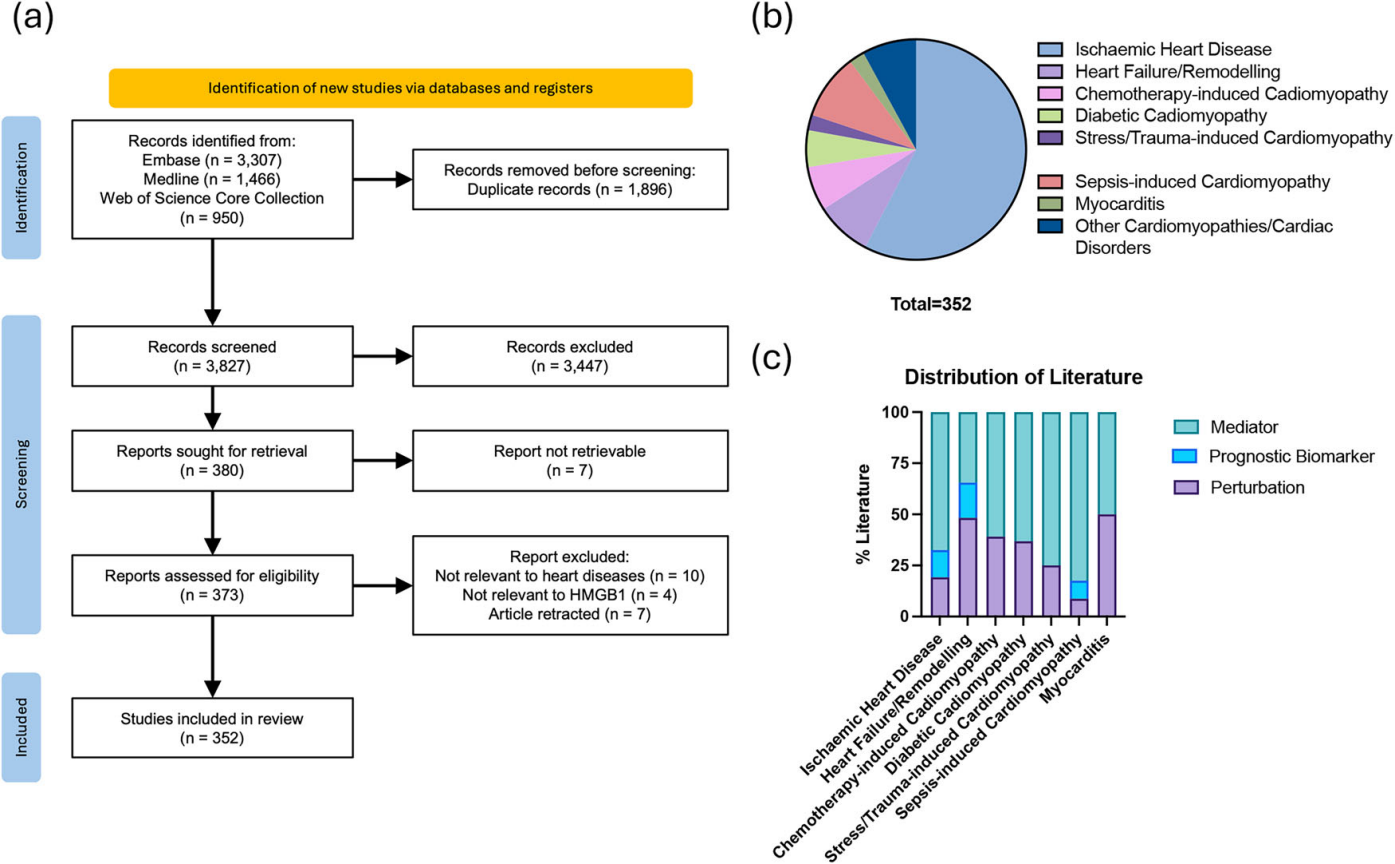
PRISMA flowchart and overview of eligible literature. **a** PRISMA flowchart showing the study selection process*. **b** Distribution of heart diseases covered in the included studies. **c** Categorisation of the literature based on study type and focus. *Created using an adaptation of a web-based tool (Haddaway et al., 2022 10.1002/cl2.1230).

Our search identified 3818 articles, with 352 meeting the eligibility criteria (Fig. 2a). These studies were categorised by heart disease type, including ischaemic heart disease (IHD), HF, myocarditis, chemotherapy-induced, sepsis-induced, trauma or stress-induced, and other cardiomyopathies and cardiac disorders (Fig. 2b). Each category was subdivided into three sub-categories: HMGB1 as a mediator (Supplementary Data 1), a prognostic biomarker, and HMGB1 perturbation (Fig. 2c).

More than half of the included studies characterised HMGB1 as a critical mediator of disease pathology (Fig. 2c). Consistently, HMGB1 levels were found to be elevated in cardiac disorders, whilst therapeutic interventions that improved clinical outcomes were associated with the downregulation of HMGB1 (Supplementary Data 1). However, most studies reported associations rather than causality. Studies reporting on HMGB1 perturbation provided mechanistic insights and others highlighted the clinical prognostic value of HMGB1 in cardiac diseases.

### Ischaemic heart disease (IHD)

IHD remains the most prevalent cardiovascular disorder globally^1^. Coronary artery occlusion, typically driven by atherosclerotic thrombosis or embolism, precipitates myocardial ischaemia, necrosis, and inflammation^53^. The consequent loss of cardiomyocytes and formation of fibrotic scar tissue drive adverse ventricular remodelling and functional decline, ultimately leading to HF^53^. During ischaemia, HMGB1 is released passively from necrotic cells or actively translocated from the nucleus to the cytoplasm and extracellular space^29–31^. Whilst there is evidence that levels of HMGB1 in the circulation may have prognostic value^54–58^, its specific pathophysiological effects in IHD remain unclear, particularly regarding the impact of subcellular location and redox state.

HMGB1 levels in the myocardium or circulation of animals were reported as elevated following ischaemic myocardial injury, with or without reperfusion, as well as intracellularly and extracellularly in cardiomyocytes subjected to hypoxia in vitro^29,34,59^. Circulating levels of HMGB1 peaked at 12 h in patients and on day 1 in rats, whilst HMGB1 mRNA expression in the infarcted myocardium peaked at day 7 post-MI in rodents^29–31^.

Human clinical studies highlighted the correlation between circulating HMGB1 levels and coronary artery disease (CAD) severity and progression, particularly in acute coronary syndromes (ACS), including ST-elevation myocardial infarction (STEMI), non-STEMI (NSTEMI), and unstable angina (UA). Elevated HMGB1 levels were associated with infarct size and left ventricular ejection fraction (LVEF)^54,55^. HMGB1 levels also correlated with key biomarkers such as high-sensitivity C-reactive protein (hs-CRP) and cardiac troponin I (cTnI)^54,56^.

HMGB1 may also serve as a prognostic marker for major cardiovascular events (MACE) and mortality post-ACS. High Gensini scores and elevated IL-6, together with higher HMGB1 levels in patients with extensive coronary lesions, were predictive of adverse outcomes, including heart-related death, infarction recurrence, HF, and stroke^57^. Higher HMGB1 levels were observed in non-survivors of STEMI^58^. Multivariate analysis confirmed the prognostic value of HMGB1, and combination with hs-CRP, cTnI, and BNP enhanced early risk stratification in UA and NSTEMI patients^56^. Additionally, higher HMGB1 levels correlated with worse cardiopulmonary function (poor exercise tolerance and low LVEF) and angiographic evidence of the extent of atherosclerotic plaques in CAD patients. Elevated levels of HMGB1 were associated with CAD development, reinforcing its potential as a biomarker for disease progression and prognosis (Supplementary Note 3).

Taken together, the evidence indicates that HMGB1 is released into the circulation and levels positively correlate with infarct size, the extent of coronary occlusion, and clinical outcomes. However, it is important to note that the ELISA-based assays used to measure circulating levels are unable to distinguish between the two redox states. The rapid oxidation together with the longer half-life of dsHMGB1 suggests that the disulfide form likely predominates in the circulation, especially at later timepoints^24,28^.

Consistent with higher circulating levels being associated with worse outcomes is evidence that exogenous administration of recombinant HMGB1 (rHMGB1) increased infarct size, and was associated with elevated proinflammatory cytokine levels (e.g., TNF, IL-6, IL-18, and IL-1β) and increased cell death in rodent models of permanent ligation (PL) and ischaemia-reperfusion (IR)^29,32–34,40,41^ (Table 1). Conversely, inhibition of HMGB1 by Box A^29,32–34^, anti-HMGB1 antibodies^35–37^, immunoprecipitation^41,60^, HMGB1 silencing using siRNA or shRNA^60–63^, or glycyrrhizin^34,40,59,64,65^ ameliorated myocardial injury (Table 1).

**Table 1:** Mechanistic insight into HMGB1 in ischaemic heart disease (detrimental effects)

Authors (Year)	Study population (Model)	Agonist HMGB1 (Supplier)	Antagonist of HMGB1	Receptors and pathways
Andrassy et al. (2008)^32^	C57Bl/6WT & RAGE^−/−^ mice (IR)	rHMGB1 (non-specified)	Box A	RAGE; JNK/ERK/ NF-κB
Cai et al. (2017)^65^	SD rats (IR)	–	Glycyrrhizin	ERK/p38/MAPK/JNK
Chen et al. (2019)^63^	SD rats (CME)	–	siRNA	–
Di Maggio et al. (2017)^21^	Human cardiac fibroblasts (Hypoxia); C57Bl/6mice (PL)	3SHMGB1 (in-house production)	–	CXCR4
Lusha et al. (2017)^60^	SD rats (IR)	–	siRNA	–
Han et al. (2024)^136^	H9c2 cells (H/R); C57Bl/6mice (IR)	–	Glycyrrhizin	–
Hu et al. (2018)^35^	NRCM (IR)	rHMGB1 (not-specified)	Anti-HMGB1	IL-17A
Hu et al. (2022)^61^	RAW264.7 macrophages (Hypoxia)	HMGB1 overexpression by transfection	siRNA	*Foxp1*
Mersmann et al. (2013)^33^	C57Bl/6 & TLR-2^−/−^ mice (IR)	rHMGB1 (eBioscience)	Box A	TLR-2
Ouyang et al. (2016)^62^	H9c2 cells (H/R); SD rats (IR)	HMGB1 overexpression by transfection	shRNA	Discoidin Domain Receptor 1 (DDR1)
Ren et al. (2020)^66^	C57Bl/6WT & TLR-4^−/−^ mice (ISO)	rHMGB1 (Sigma-Aldrich)	–	TLR-4
Tian et al. (2019)^41^	C57Bl/6WT & RAGE^−/−^ & TLR-9^−/−^ mice (IR)	rHMGB1 (Thermo Fisher)	Immunoprecipitation	RAGE/TLR-9
Volz et al. (2010)^29^	C57Bl/6WT & RAGE^−/−^ mice (IR)	rHMBG1 (not-specified)	Box A	RAGE
Xia et al. (2018)^36^	SD rats (IR)	–	Anti-HMGB1	–
Xu et al. (2011)^34^	NMCM (A/R); C57Bl/6 & TLR-4^−/−^ mice (IR)	rHMGB1 (Abcam)	Box-A	TLR-4/JNK/P65
Xue et al. (2019)^37^	SD rats (IR)	–	Anti-HMGB1	TLR-4/MyD88/NF-κB
Yuan et al. (2021)^59^	SD rats (CME)	–	Glycyrrhizin	TLR-4/NF-κB
Zhai et al. (2012)^40^	SD rats (IR)	rHMGB1 (not-specified)	Glycyrrhizin	pJNK/Bax
Zhao et al. (2023)^127^	Human microvascular endothelial cells (STEMI patients)	rHMBG1 (HMGBiotech)#	Box A	TLR-4
Zhu et al. (2024)^64^	H9c2 cells (H/R); SD rats (IR)	–	Glycyrrhizin	TLR-4/GPX4

Mechanistically, these pro-inflammatory effects result from the engagement of TLRs and RAGE, suggesting that the detrimental effects of HMGB1 are due to the disulfide form. Several studies reported that rHMGB1 exacerbated myocardial damage following IR via JNK/ERK/NF-κB signalling pathways^32,34,40^. The deleterious effects of rHMGB1 were significantly reduced in transgenic mice lacking TLR-2, TLR-4, TLR-9, or RAGE^29,32–34,41,66^. Consistent with these findings, anti-HMGB1 downregulated inflammation by limiting dendritic cell activation via the TLR-4/MyD88/NF-κB axis in an IR model in rats^37^. Furthermore, glycyrrhizin-mediated HMGB1 inhibition protected the myocardium from cell death (apoptosis and ferroptosis) in IR models in rats by suppressing TLR-4 signalling^59,64^ and inhibiting the ERK/p38 MAPK/JNK pathways^65^. HMGB1 has been shown to transcriptionally regulate *FOXP1*, a tumour suppressor gene which can modify the inflammatory response in macrophages following MI. Hypoxia resulted in increased proinflammatory cytokine expression by RAW264.7 macrophages, with concomitant increase in HMGB1 and decrease in *Foxp1* expression^61^. This indicates that intracellular HMGB1 in macrophages and extracellular dsHMGB1 are both deleterious in IHD.

In contrast, other studies have reported that HMGB1 plays a beneficial role in MI (Table 2). HMGB1 inhibition was reported to disrupt reparative processes following MI, and animals treated with anti-HMGB1 antibodies developed larger infarcts and impaired cardiac function^30,44^. Furthermore, several studies have reported that exogenous administration can also be beneficial. Exogenous administration of rHMGB1 reduced infarct size, oxidative stress, and expression of proinflammatory cytokines in rodent MI models^45–47,67–73^. Intramyocardial injection of rHMGB1 four hours after left anterior descending (LAD) ligation significantly improved cardiac function and reduced both infarct size and fibrosis in murine and ovine models of MI^45–47^.

**Table 2:** Mechanistic insight into HMGB1 in ischaemic heart disease (beneficial effects)

Authors (Year)	Study population (Model)	Agonist HMGB1 (Supplier)	Antagonist of HMGB1	Receptors and pathways
Abarbanell et al. (2011)^67^	SD rats (Langendorff)	rHMGB1 (Sigma-Aldrich)	–	–
Bauzá et al. (2019)^47^	Corriedale sheep (PL)	rHMGB1 (HMGBiotech)#	–	–
Di Maggio et al. (2017)^21^	Human cardiac fibroblasts (Hypoxia); C57Bl/6mice (PL)	frHMGB1 (In-house production)	–	CXCL12/CXCR4
Foglio et al. (2017)^73^	C57Bl/6mice (PL)	rHMGB1 (In-house production)	–	TLR-9/RAGE; AMPK/Akt/mTOR
He et al. (2013)^46^	SD rats (PL)	rHMGB1 (not-specified)	–	–
Kitahara et al. (2008)^31^	WT&HMGB1-TG mice (PL)	Cardiomyocyte-specific HMGB1 overexpression (α-MHC promoter)	–	–
Kohno et al. (2009)^30^	Human (STEMI); Wistar rats (PL)	–	Anti-HMGB1	–
Limana et al. (2005)^45^	C57Bl/6mice (PL)	rHMGB1 (In-house production)	Box A	c-kit^+^ cardiac stem cells
Limana et al. (2010)^130^	Human (MI); C57Bl/6mice (PL)	–	–	c-kit^+^ cardiac stem cells
Limana et al. (2013)^74^	C57Bl/6mice (PL)	rHMGB1 (In-house production)	–	Notch
Liu et al. (2019)^72^	Human (DCM); C57Bl/6WT&TLR-9^-/-^ mice (PL)	rHMBG1 (R&D)	–	TLR-9
Liu et al. (2024)^137^	Cardiac endothelial cells (Hypoxia)	–	siRNA	YAP1
Lozano et al. (2022)^138^	hiPSCs, CMs, HUVECs, hCFs (Hypoxia)	HMGB1 (Stem-cell derived EVs)	–	–
Nakamura et al. (2015)^48^	C57Bl/6WT & HMGB1-TG mice (PL)	Cardiomyocyte-specific HMGB1 overexpression (α-MHC promoter)	–	–
Oozawa et al. (2008)^44^	Wistar Rats (IR)	–	anti-HMGB1	Norepinephrine
Rossini et al. (2008)^139^	hCFs and mouse CSCs (Hypoxia)	HMGB1(HMGBiotech)#	–	RAGE
Yao et al. (2016)^71^	Wistar rats (IR)	rHMGB1 (R&D)	–	HIF-1α/PI3K/AKT
Zhou et al. (2012)^68^	SD rats (PL)	rHMGB1 (not-specified)	–	β-catenin/Dishevelled-1
Zhou et al. (2017)^70^	Wistar Rats (IR)	rHMGB1 (Sino Biological Inc.)	–	HIF-1α/p38 MAPK
Zhou et al. (2020)^69^	Wistar rats (IR)	rHMGB1 (not-specified)	–	PI3K/Akt

#HMGBiotech specifies the redox forms of HMGB1 on their website. However, these studies did not include the catalogue number or specify the redox forms used.

*PL* permanent ligation, *HMGB1-TG* transgenic mice with cardiac-specific overexpression of HMGB1, *DCM* dilated cardiomyopathy, *hiPSCs* human induced pluripotent stem cells, *CMs* cardiomyocytes, HUVECs human umbilical vein endothelial cells, *hCF* human cardiac fibroblasts, *MI* myocardial injury, *IR* ischaemia-reperfusion, *EVs* extracellular vesicles, *CSCs* cardiac stem cells, *frHMGB1* fully-reduced HMGB1, *3SHMGB1* 3-serine HMGB1, *STEM*I ST-elevation myocardial infarction, H/R hypoxia/reoxygenation, CME coronary microembolisation, *NMCM* neonatal mouse cardiomyocyte, *A/R* anoxia-reoxygenation, *NRCM* neonatal rat cardiomyocyte, *WT* wild-type, *ISO* isoproterenol.

Several mechanisms were proposed to explain these findings, including cardioprotection, angiogenesis, and cardiac regeneration. The cardioprotective effects of HMGB1 were mediated through upregulation of HIF-1α, p38 MAPK inhibition, and activation of the PI3K/AKT pathway, leading to increased VEGF protein levels, an effect that was abolished by PI3K inhibitors^69–71^. Additionally, HMGB1 promoted autophagy through AMPK-dependent inhibition of mTOR and p-AKT, reduced apoptosis^73^ and improved cardiac remodelling by activating Wnt/β-catenin signalling and reducing collagen deposition^68^. Angiogenesis is essential for the repair of damaged myocardium and maintaining endothelial integrity. rHMGB1 enhanced angiogenesis in the border zone around the infarcted area^45–47,72^, with a concomitant upregulation of VEGF expression^69^. These effects were absent in TLR-9^−^^/−^ mice^72^, suggesting angiogenesis is promoted, at least in part, through TLR-9 signalling. rHMGB1 treatment was reported to enhance cardiac regeneration by increasing the number of proliferative c-kit⁺/Ki67⁺ cells and upregulating early cardiomyogenic transcription factors, including *MEF2C* and *NKX2-5*, thereby promoting the renewal and replenishment of cardiomyocytes and cardiac progenitors^45–47^. Transcriptomic profiling at day 3 post-treatment revealed activation of regenerative processes via the Notch signalling pathway, with a 35% and 58% increase in Notch intracellular cytoplasmic domain expression in cardiomyocytes and cardiac progenitor cells, respectively^74^. However, cardiac regeneration in adult mammals through c-kit^+^ cells has now been largely discredited^75^.

Although the improved repair following exogenous administration is likely to be attributed to the fully reduced form, only one study clearly defined the redox state of the exogenous rHMGB1 used as frHMGB1. The authors showed that intramyocardial injection of frHMGB1 4 h after PL improved ejection fraction and other parameters of left ventricular function at one week, reduced fibrosis and left ventricle dilatation at 4 weeks via CXCL12/CXCR4 signalling^21^. Interestingly, a non-oxidisable HMGB1 variant (3SHMGB1), engineered by substituting the three cysteine residues (C23, C45, and C106) in frHMGB1 with serine, exacerbated adverse remodelling and impaired cardiac function following MI, partly by abolishing angiogenic effects and promoting chemotaxis of cardiac fibroblasts by signalling via CXCR4 independently of CXCL12^21^. These findings underscore the critical role of defining the redox state, structure, and ligand–receptor interaction of HMGB1 when considering its effects in MI.

Interestingly, the overexpression of HMGB1 yielded beneficial effects comparable to those observed with exogenous administration. Mice with cardiomyocyte-specific HMGB1 overexpression had a 30% reduction in infarct size and 90% improvement in survival rate compared to wild-type controls following MI, with enhanced angiogenesis in the border zone^31^. Plasma HMGB1 levels were also elevated in these mice^31^. This finding stands in contrast to prior reports where elevated circulating HMGB1 was consistently correlated with worse clinical outcomes. GFP-labelled bone marrow cells injected into irradiated recipient mice before MI surgery showed increased chemotaxis and differentiation of bone marrow-derived endothelial progenitor cells in the border zone of mice overexpressing HMGB1^48^. It is unclear whether the beneficial effects of transgenic upregulation were due to higher intracellular levels of HMGB1, increased release of frHMGB1 following cell injury, or both.

In summary, the role of HMGB1 in IHD remains controversial. However, current evidence suggests that the redox state and subcellular location may act as critical determinants of its function. Effects of exogenous administration of HMGB1 are explained by downstream signalling. Only one study identified the redox form of the HMGB1 used as frHMGB1 and found that it signalled via CXCL12 and CXCR4^21^. 3SHMGB1 signals directly via CXCR4 independently of CXCL12 and resulted in increased fibrosis^21^. HMGB1 constructs that signalled via TLRs or RAGE were deleterious^29,32–34,41,66^.

Distinct from MI, which is characterised by direct ischaemic necrosis, conditions such as sepsis-induced cardiomyopathy, myocarditis, and trauma- or stress-induced cardiomyopathy do not primarily involve acute ischaemic cell death. Instead, HMGB1 upregulation in these pathologies appears to be a response to stress-related stimuli, such as excessive inflammation or catecholamine surges. In these contexts, the exogenous administration of HMGB1 consistently results in the exacerbation of myocardial dysfunction. It is likely that the effects were due to dsHMGB1, as they are abrogated by the knockout of RAGE^76^. Interestingly, cardiomyocyte-specific conditional knockout of HMGB1 was detrimental^77^, suggesting that intracellular HMGB1 in these cells is protective.

### Sepsis-induced cardiomyopathy

Sepsis-induced cardiomyopathy is a reversible form of cardiac dysfunction driven by the systemic cytokine storm associated with severe infection^78^. HMGB1 has been identified as a potential prognostic biomarker for evaluating therapeutic efficacy and monitoring disease progression^79–81^. Circulating levels inversely correlated with outcomes, and exogenous administration of rHMGB1 resulted in acute myocardial depression and impaired contractility^82^.

Clinical trials of Sivelestat and vitamin C reported improved cardiac outcomes and survival rates associated with lower systemic HMGB1 levels in patients with septic cardiomyopathy^79,80^. HMGB1 may also be useful as part of a biomarker panel to assess the risk of left ventricular diastolic dysfunction (LVDD) in septic patients when combined with cTnI and BNP, with a sensitivity of 90% and specificity of 94%^81^.

rHMGB1 administration to ex vivo lipopolysaccharide (LPS)-treated rat hearts induced a transient negative inotropic effect, reducing LVEF, LV +d*P*/d*t*max, and LV −d*P*/d*t*min, whilst increasing left ventricular end-diastolic pressure. This occurred within 1–10 min of perfusion, and the effects were reversed upon rHMGB1 withdrawal, suggesting that acute HMGB1 exposure transiently impaired cardiac contractility during endotoxemia^83^. Inhibition of HMGB1/TLR-4 signalling with glycyrrhizin reduced oxidative stress and cytokine release in LPS-stimulated cardiomyocytes and improved cardiac function in mice exposed to LPS^82^. Additionally, LPS-induced HMGB1 expression in cardiomyocytes via TLR-4-mediated PI3Kγ phosphorylation. Genetic deletion or pharmacological inhibition of PI3Kγ significantly reduced LPS-induced HMGB1 expression and secretion, thereby ameliorating depression of myocardial contractility^84^.

Cardiomyocyte-specific conditional deletion of HMGB1 (Hmgb1^f/f^ TgCre^/+^) exacerbated myocardial injury in a model of Coxsackievirus B3 virus (CVB3)-induced myocarditis. This exacerbation was associated with increased susceptibility of HMGB1-deficient cardiomyocytes to early apoptosis via the p53-mediated Bax mitochondrial pathway, suggesting that intracellular HMGB1 in cardiomyocytes protects against the development of myocarditis^77^. Collectively, these data suggest that intracellular HMGB1 in cardiomyocytes is protective and that the detrimental effects were likely due to dsHMGB1 in sepsis-induced cardiomyopathies.

### Autoimmune and stress-induced cardiomyopathies

Autoimmune myocarditis is associated with inflammation and stress, with concomitant upregulation of HMGB1 levels in preclinical models^85^. Administration of exogenous rHMGB1 resulted in increased myocardial fibrosis in murine experimental autoimmune myocarditis (EAM) through upregulation of MMP1/2 and TIMP1 in cardiac fibroblasts and myofibroblasts via the PKCβ/Erk1/2 signalling pathway^86^. Inhibition of HMGB1 with a neutralising antibody reduced cardiac fibrosis in EAM, accompanied by a reduction in immune infiltrates, including the autoimmunity-contributing Th17^+^ cells^85^. Inhibition of HMGB1 with an antibody or glycyrrhizin attenuated EAM, and RAGE^−/−^ mice were protected from the development of EAM^76^. Given that dsHMGB1 signals via RAGE, these findings suggest that targeting the dsHMGB1–RAGE axis may be of benefit in autoimmune myocarditis.

Stress-induced cardiomyopathy is an acute cardiac dysfunction precipitated by a massive surge of catecholamines following severe physical or emotional trauma^87^. It is characterised by transient apical ballooning of the left ventricle, mimicking MI, but is typically reversible^87^. Haemorrhagic shock and resuscitation resulted in myocardial injury in animals and were associated with upregulation of HMGB1^88,89^. Administration of anti-HMGB1 reduced cardiomyocyte apoptosis, downregulated TLR-4 and HMGB1 in the myocardium, and decreased pro-inflammatory cytokine (IL-1β, IL-6, TNF) levels^88,89^.

Unlike MI, which is characterised by direct cellular necrosis and the passive release of frHMGB1, acute cardiomyopathies result from cytokine or catecholamine storms. These conditions generate a pro-oxidative environment that facilitates the secretion of HMGB1 by immune and stromal cells. In this context, HMGB1 is likely to be present predominantly in the pro-inflammatory dsHMGB1, consistent with inflammatory pathology mediated through TLRs^82,84,88,89^ and RAGE^76^ signalling. Passive release of frHMGB1 from injured cardiomyocytes acts as a chemokine to recruit more immune cells, which in turn actively secrete dsHMGB1, thereby amplifying local inflammation in a feed-forward manner. Therefore, inhibition of HMGB1 is likely to be beneficial in acute cardiomyopathies.

### Heart failure/adverse remodelling/hypertrophy

The prevalence of HF and chronic heart disease is rising, driven by an ageing population, increased comorbidities^90^, and improved survival rates following the implementation of early revascularisation strategies for MI^5^. In this chronically stressed myocardial environment, HMGB1 may play a role distinct from its function in acute cardiac disorders. However, as in the acute disease setting, there are conflicting accounts on its role in chronic disease, again likely explained by the subcellular location and redox state.

HMGB1 has been proposed as a prognostic biomarker for HF, aiding in monitoring disease progression and treatment outcomes. Levels correlated with established markers of HF, including NT-proBNP, and functional parameters such as left ventricular end diastolic volume (LVEDV), left ventricular end systolic volume (LVESV), LVEF, left ventricular mass index, and myocardial wall stress^91–94^. Circulating HMGB1 levels were elevated in HF patients and correlated with disease severity^92,93^. Patients with advanced HF (New York Heart Association [NYHA] stages III/IV) had significantly higher HMGB1 levels compared to those with less severe disease (NYHA I/II) or healthy controls^92^. Multivariate analysis confirmed HMGB1 as an independent predictor of disease severity and adverse outcomes, including necessity for heart transplantation or death^92^.

HMGB1 levels were also significantly increased in patients with pulmonary arterial hypertension (PAH) due to congenital heart disease and correlated with pulmonary arterial pressure and pulmonary vascular resistance. After 6 months of sildenafil therapy, HMGB1 levels in PAH patients decreased, with concomitant reduction in right atrial and ventricular diameters and pulmonary artery pressure^95^. However, HMGB1 levels can also be influenced by comorbidities. Patients with concurrent conditions had higher HMGB1 levels compared to those with HF alone^93^, highlighting the need to consider all underlying conditions when interpreting its prognostic value.

Most studies suggested that HMGB1 overexpression, particularly within the nuclei of cardiomyocytes or immune cells, was protective^49,50,96,97^. Exogenous HMGB1 administration demonstrated inconsistent results^42,43,98,99^, presumably depending on the redox state, which was not defined in any of the articles retrieved.

In a mouse model of heart failure with preserved ejection fraction (HFpEF), administration of an HMGB1 inhibitor, glycyrrhizin, resulted in attenuation of fibrosis and diastolic dysfunction, although there was no improvement in systolic function^38^. Mechanistically, there was decreased neutrophil infiltration and neutrophil extracellular traps, suggesting that the functional improvement was due to reduced inflammation^38^ (Table 3).

**Table 3:** Mechanistic insights into HMGB1 in heart failure and remodelling (detrimental effects)

Authors (Year)	Study population (Model)	Time of interventions (in vivo route)	Agonist HMGB1 (Supplier)	Antagonist HMGB1	Receptors and Pathways
Lin et al. (2016)^102^	NRCMs^a^; WT & RAGE^−/−^ mice (TAC)	–	rHMGB1 (Sigma-Aldrich)^b^	–	RAGE
Su et al. (2021)^100^	NMCMs^a^	–	rHMGB1 (Sigma-Aldrich)^b^	–	14-3-3η/PI3K/Akt/NFAT3
Zhang et al. (2016)^42^	NRCMs (Mechanical stress); C57Bl/6J mice (TAC)	Before TAC (i.m.c.)	rHMGB1 (HMGBiotech)^c^	Box-A	–
Zhang et al. (2019)^101^	H9c2 cells & NRCMs (Mechanical stress)	–	rHMGB1 (HMGBiotech)^b,^^c^	–	RAGE; ERK1/2
Zhang et al. (2020)^43^	C57Bl/6J mice (TAC)	Before TAC (i.m.c.)	rHMGB1 (HMGBiotech)^c^	–	–
Zhang et al. (2022)^38^	Patients (HFpEF); C57Bl/6mice (HFpEF)	1 day post TAC (i.p.)	–	Glycyrrhizin	–

^a^Co-culture with rHMGB1 without additional injury.

^b^In vitro assay only.

^c^HMGBiotech specifies the redox forms of HMGB1 on their website. However, these studies did not include the catalogue number or specify the redox forms used.

HMGB1 injected into the myocardium at the time of transverse aortic constriction (TAC) surgery exacerbated cardiac remodelling and reduced cardiac function over four weeks^42,43^. This was associated with upregulation of expression of collagen type I, TGF-β, and MMP2 in the myocardium^43^. Mechanistically, rHMGB1-induced stress protein expression and nuclear translocation of NFAT3, promoting hypertrophy via the PI3K/AKT/NFAT signalling pathway, which was reversed by PI3K inhibition^100^. The deleterious effects of intramyocardial injection of HMGB1 in a TAC model were partially antagonised by Box A^42^. Whilst the redox state of the rHMGB1 used was not specified, we can surmise that it was dsHMGB1.

However, the role of RAGE in mediating these processes remains unclear. HMGB1 exacerbated mechanical stress-induced cardiomyocyte hypertrophy in vitro by activating ERK1/2 through RAGE^101^. Nevertheless, despite elevated HMGB1 levels in the myocardium, RAGE^−/−^ mice did not exhibit improved cardiac function compared to wild-type controls 4 weeks post-TAC. Furthermore, administration of neutralising RAGE antibody did not inhibit the effects of exogenous HMGB1-induced cardiomyocyte hypertrophy^102^. Whilst these deleterious effects in TAC-induced heart failure can likely be attributed to dsHMGB1, targeting RAGE alone may be insufficient, as dsHMGB1 can also activate downstream inflammatory pathways via TLR-2 and TLR-4. TLR-4^−/−^ mice subjected to TAC showed reduced cardiac hypertrophy compared to wild-type controls^103^.

Other studies have reported beneficial effects of exogenous administration of HMGB1 in heart failure models (Table 4). Intramyocardial injection of HMGB1 administered 2–3 weeks following PL of the LAD in rodent models of HF significantly improved cardiac function. A 6–10% increase in LVEF was observed four weeks post-injection^98,99,104^. There was a concomitant increase in left ventricular wall thickness and reduced collagen deposition^98,99^. Cardiac regeneration and neovascularisation were suggested as potential mechanisms, evidenced by increases in new cardiomyocytes derived from c-kit^+^ cells and higher vascular density^98^. Others suggested HMGB1 modulated inflammation by reducing infiltration of inflammatory cells in the border zone, particularly OX62^+^ dendritic cells, via activation of ERK1/2^104^.

**Table 4:** Mechanistic insights into HMGB1 in heart failure and remodelling (beneficial effects)

Authors (Year)	Study population (Model)	Time of interventions (in vivo route)	Agonist HMGB1 (Supplier)	Antagonist HMGB1	Receptors and Pathways
Funayama et al. (2013)^49^	Patients (HF); NRCMs (ET-1&Ang II); WT & HMGB1-Tg mice (TAC)	–	Cardiomyocyte-specific HMGB1 overexpression (α-MHC promoter)	siRNA	–
Goto et al. (2020)^105^	SD rats (PL)	2wk post MI (i.v.)	HMGB1 Box-A fragment (StemRIM)	–	–
He et al. (2013)^99^	SD rats (PL)	3wk post MI (i.m.c.)	rHMGB1 (R&D)	–	TGF-β-Smad
Limana et al. (2011)^98^	C57Bl/6mice (PL)	3wk post MI (i.m.c.)	rHMGB1 (HMGBiotech)^a^	–	c-kit+ cardiac stem cells; miR-206 and inhibition of TIMP-3
Li et al. (2019)^96^	NRCMs (ang-II, PE, ISO); SD rats (3AB, AAC)	–	HMGB1 overexpression by transfection (plasmid pcDNA3.1-6Flag-HMGB1)	–	PARP1-mediated nuclear HMGB1 translocation
Takahashi et al. (2008)^104^	SD rats (PL)	3wk post MI (i.m.c.)	rHMGB1 (R&D)	–	ERK1/2
Takahashi et al. (2019)^50^	Patients (HF); NRCMs (Ang II); WT & HMGB1-Tg mice (TAC)	–	Cardiomyocyte-specific HMGB1 overexpression (α-MHC promoter)	siRNA	–
Yang et al. (2022)^97^	LysM-HMGB1^−/−^ mice (TAC)	–	–	Myeloid-specific HMGB1 deletion	TGF-β-Smad

^a^HMGBiotech specifies the redox forms of HMGB1 on their website. However, these studies did not include the catalogue number or specify the redox forms used.

*HF* heart failure, *NRCMs* neonatal rat cardiomyocytes, *ET-1* endothelin-1, *Ang II* angiotensin II, *HMGB1-TG* transgenic mice with cardiac-specific overexpression of HMGB1, *TAC* thoracic transverse aortic constriction, *PL* permanent ligation, *PE* phenylephrine, *ISO* isoproterenol, *3AB* 3-aminobenzamide, *AAC* abdominal aortic constriction, *KO* knock-out, *NMCM* neonatal mouse cardiomyocyte, *HFpEF* heart failure with preserved ejection fraction, *WT* wild-type, *i.m.c.* intra-myocardial injection, *i.v.* intravenous injection, *i.p.* intraperitoneal injection.

Most studies have utilised Box A as a competitive antagonist to HMGB1. However, one study reported that systemic administration of Box A fragment in MI-induced HF resulted in 15% improvement of LVEF as a result of enhanced angiogenesis and reduced fibrosis, mediated by the recruitment of PDGFRα^+^ mesenchymal cells from the bone marrow to the border zone^105^.

Subcellular location is critical to the function of HMGB1 in HF, particularly within the nucleus. Preserving nuclear HMGB1 by limiting its export to the cytoplasm has been shown to protect against the development of HF. In vitro stressing of rat neonatal cardiomyocytes resulted in acetylation of nuclear HMGB1 and export to the cytoplasm^49^. In the human failing heart, levels of nuclear HMGB1 negatively correlated with DNA damage. In these samples, nuclear HMGB1 expression was downregulated as the protein translocated from the nucleus to the cytoplasm^50^.

Overexpression of poly(ADP-ribose) polymerase 1 (PARP1) promoted nuclear export and extracellular secretion of HMGB1, contributing to pathological cardiac hypertrophy, whereas PARP1 inhibitors mitigated these effects^96^. Cardiomyocyte-specific HMGB1 overexpression, under the control of an α-MHC promoter, attenuated hypertrophy and improved cardiac function in TAC-induced heart failure and angiotensin II-induced hypertrophy mouse models, protecting the heart from pathological hypertrophy by reducing DNA damage^49,50^.

Furthermore, mice with myeloid-specific HMGB1 deletion had worse cardiac function in a TAC-induced heart failure model compared to wild-type controls^97^. Loss of HMGB1 in macrophages was associated with an increased ratio of M1/M2 macrophages and elevated inflammatory cytokines, indicating a shift towards proinflammatory macrophage differentiation in the myocardium. Collectively, these data suggest that increased intracellular levels of HMGB1, particularly intranuclear, are protective.

In the context of HF and adverse remodelling, the role of HMGB1 remains unclear and less well-characterised compared to MI. The impact of extracellular HMGB1 administration is particularly difficult to interpret, as none of the studies have defined the redox state of HMGB1 used^42,43,98,99,104^. However, based on the downstream receptors, it can be deduced that deleterious effects in models of heart failure can likely be attributed to dsHMGB1. From the available evidence, it is not possible to use a similar approach to deduce that the beneficial effects were due to frHMGB1. In contrast, the upregulation of nuclear HMGB1 expression appears consistently protective^49,96^, underscoring the critical effect of subcellular location. Mechanistically, whilst HMGB1-induced angiogenesis is well-supported, other proposed reparative pathways, such as the differentiation of c-kit^+^^98^ or bone marrow-derived cells^105^ to replace dead myocardium, have been largely discounted^75,106^, with evidence suggesting that the injection of killed cells can yield similar non-specific effects. Consequently, resolving these discrepancies requires a rigorous definition of the redox state and reappraisal of the mechanism of action.

### Diabetic cardiomyopathy

Diabetes is associated with the development of cardiomyopathy through exacerbation of inflammation, endoplasmic reticulum stress and cellular death, leading to increased fibrosis and cardiac dysfunction^39,107–109^. There was concurrent upregulation of cytoplasmic translocation of nuclear HMGB1 in cardiomyocytes^39,107–109^.

Metabolic stress induced by a high-fat, high-glucose diet in mice triggered the expression of CCAAT/enhancer-binding protein homologous protein (CHOP), a transcriptional regulator of cardiac HMGB1. Mice treated with a high-fat and high-glucose diet showed increased expression of acetylated HMGB1 in cardiomyocytes. Exogenous administration of rHMGB1 promoted M1 macrophage polarisation in vitro, especially in media with high fat and high glucose. Treatment with glycyrrhizin improved cardiac function and reduced immune infiltration in the myocardium of mice with diabetic cardiomyopathy^107^. Inhibition of HMGB1 by shRNA or neutralising antibody downregulated the expression of MMP-2 and MMP-9 in cardiac fibroblasts under high-glucose conditions. This inhibition was associated with a reduction in hyperglycaemia-induced activation of E26 transformation-specific-1 (Ets-1) through ERK1/2 signalling. Inhibiting Ets-1 also reduced apoptosis in the myocardium of mice with diabetic cardiomyopathy. Collectively, these findings indicate that HMGB1 inhibition reduces apoptosis and MMP expression in cardiac fibroblasts via the ERK-dependent Ets-1 pathway^39^.

Exogenous rHMGB1 promoted cardiac fibroblast proliferation and migration under conditions of normal glucose, comparable to cells incubated in high glucose^109^. High glucose-induced upregulation of HMGB1 in cardiomyocytes was associated with a downregulation of IL-33 and an increase in collagen production by cardiac fibroblasts^110^. Administration of Box A or exogenous IL-33 reduced collagen deposition and attenuated cardiac dysfunction in diabetic mice. Additionally, TLR-4^−/−^ diabetic mice exhibited higher myocardial HMGB1 expression without a corresponding increase in fibrosis and downregulation of IL-33 in the fibroblasts, suggesting that HMGB1 regulates fibrosis through the TLR-4/IL-33 axis^110^.

Taken together, the data show that diabetic cardiomyopathy is associated with elevated levels of cytoplasmic HMGB1, leading to ER stress and apoptosis. rHMGB1 enhanced M1 polarisation, inflammation, and fibrosis, whilst inhibition of HMGB1 improved function. Based on the downstream signalling pathways (TLR-4), it is likely that the deleterious effects of HMGB1 in diabetic cardiomyopathy can be attributed to the disulfide form.

### Chemotherapy-induced cardiomyopathy

Chemotherapy agents induced cellular stress and damage, with concomitant upregulation of HMGB1, leading to activation of programmed cell death^111–116^. The majority of studies showed that antagonising HMGB1 reduced cell death.

HMGB1 has been reported to play a pivotal role in cell death associated with chemotherapy-induced cardiotoxicity and cardiomyopathy, whilst antagonising HMGB1 has been shown to be beneficial. Inhibition, including administration of Box A, silencing of HMGB1 with shRNA, or TLR-4 knock-out, resulted in reduced apoptosis of cardiomyocytes exposed to doxorubicin^111^. Silencing HMGB1 or inhibition with glycyrrhizin downregulated autophagy markers LC3II and p62, leading to improved cardiomyocyte viability^112,113^. Inhibition of HMGB1 attenuated maladaptive autophagy through downregulation of the Akt/mTOR pathway^112^. Moreover, overexpression of Yes-Associated Protein (YAP)/transcriptional coactivator downregulated HMGB1 expression, suggesting a regulatory role for YAP^113^.

Ferroptosis has also been implicated in doxorubicin-induced cardiomyopathy. Administration of the iron chelator, dexrazoxane, attenuated doxorubicin-induced cardiotoxicity^115,116^. Overexpression of HMGB1 reduced the efficacy of dexrazoxane by promoting ferroptosis and oxidative stress in doxorubicin-induced cardiotoxicity, whereas silencing HMGB1 had the opposite effect^115^. However, the authors induced HMGB1 expression via intravenous lentiviral delivery rather than using a cardiac-specific approach. This likely resulted in systemic overexpression across multiple cell types, thereby complicating the interpretation of the data. HMGB1 promoted inflammation during ferroptosis via immune cell activation. Depletion of neutrophils reduced the formation of neutrophil extracellular traps (NETs), downregulated HMGB1 expression, and attenuated ferroptosis, thereby improving cardiac function in doxorubicin-induced cardiomyopathy. The administration of rHMGB1 partially reversed the effect of neutrophil depletion through the YAP/TLR-4 axis^116^, suggesting that the effects were due to the disulfide form.

Dasatinib, a tyrosine kinase inhibitor used for treating chronic myeloid leukaemia, has been associated with significant cardiovascular toxicity, mainly through the induction of necroptosis in cardiomyocytes via intracellular activation of HMGB1. Genetic deletion of HMGB1 through CRISPR-Cas9 knockout increased the survival of cardiomyocytes exposed to dasatinib^114^.

Mice with cardiac-specific (α-MHC promoter) overexpression of HMGB1 exhibited reduced doxorubicin-induced cardiotoxicity compared to wild-type controls. Silencing HMGB1 in cardiomyocytes decreased mitochondrial membrane potential, which was partially restored with HMGB1 transfection. HMGB1 promoted cardiomyocyte survival by upregulating heat shock protein beta-1 (HSPB1) through activation of the heat shock element promoter. Silencing HSPB1 and heat shock factor 2 abrogated the protective effects of HMGB1. The results show that overexpression of HMGB1 in cardiomyocytes protected against doxorubicin-induced cardiotoxicity by restoring mitochondrial function through modulation of HSPB1^117^.

### Other cardiomyopathies/cardiac disorders

HMGB1 has been shown to exert detrimental effects in congenital dilated cardiomyopathy, metabolism-, ageing-, cancer-associated cardiomyopathy and arrhythmias. This literature is summarised in Supplementary Note 4.

The majority of studies suggest that HMGB1 exerts a detrimental role in the progression of chronic heart disease, as its inhibition attenuates cellular stress and damage, largely via TLR-4 signalling, suggesting that these effects are mediated by dsHMGB1^110,111,116^. Although cardiac-specific overexpression of HMGB1 has generally demonstrated protective effects in doxorubicin-induced cardiotoxicity^117^, other studies report that HMGB1 overexpression that is not restricted to cardiomyocytes can induce cell death via ferroptosis and oxidative stress^115^. Furthermore, the knockout of HMGB1 has been shown to improve the survival of cardiomyocytes treated with dasatinib^114^. Taken together, the studies on cardiomyopathies suggest that the role of HMGB1 in cardiomyopathies depends on the redox state, cellular location, as well as disease context.

## Discussion

**Fig. 3:**
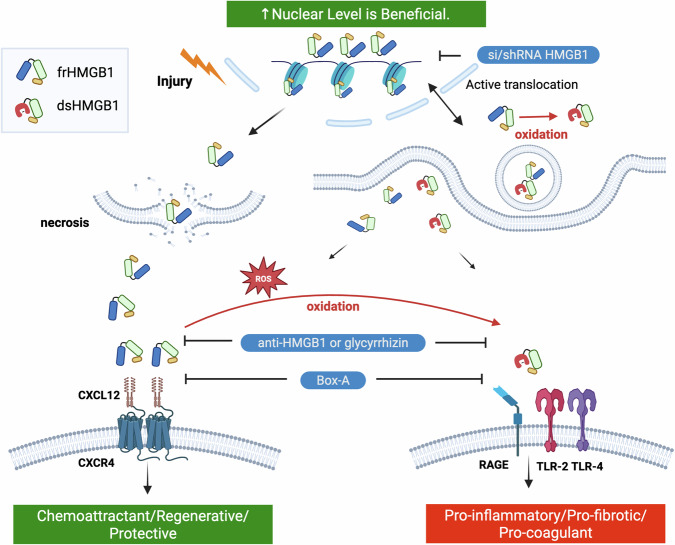
Schematic summary of HMGB1 signalling in the myocardium. HMGB1 is a non-histone chromatin-binding protein in the nucleus. Under stress conditions or following injury, HMGB1 can be passively released into the extracellular space or transported to the cytoplasm and then actively secreted into the extracellular space by packing into intracellular vesicles (lysosomes or autophagosomes) and fusing with the cytoplasmic membrane^12^. HMGB1 can undergo post-translational modifications (PTM) affecting nuclear localisation signals (NLS) and nuclear export signal (NES) to facilitate translocation of nuclear HMGB1 to the cytoplasm^11,12^. These PTMs include acetylation, phosphorylation, methylation, N-glycosylation, and oxidation. The redox state of HMGB1 modulates function both intra- and extracellularly^25,118,119^. Conformational changes as a result of the disulfide bridge between C23 and C45 alter the affinity of HMGB1 for intracellular shuttling proteins and extracellular receptors. Intracellularly, upregulation of nuclear HMBG1 has been shown to be beneficial. The function of cytoplasmic HMGB1 depends on post-translational modifications and is not fully understood. Extracellular HMGB1 acts as an alarmin. frHMGB1 forms a heterocomplex with CXCL12 and signals via CXCR4 to promote chemotaxis and tissue repair. dsHMGB1 signals via TLR-2, TLR-4, and RAGE to promote the release of pro-inflammatory cytokines. The dsHMGB1–RAGE axis also activates platelets. Various strategies have been explored to inhibit HMGB1, including Box A, glycyrrhizin, anti-HMGB1 antibodies, or small interfering RNA. Box A acts as a competitive inhibitor of HMGB1, whereas glycyrrhizin or neutralising antibodies bind to the HMGB1. However, none of these approaches can target a specific redox form of HMGB1. Created in BioRender. Mao, S-H. (2026) https://BioRender.com/scr20ip frHMGB1 fully reduced HMGB1, dsHMGB1disulfide HMGB1.

The multifaceted role of HMGB1 is determined by subcellular location and redox state. The redox state dictates the downstream signalling—frHMGB1 forms a heterocomplex with CXCL12 and signals via CXCR4^19–23^, whilst dsHMGB1 signals via RAGE, TLR-2, and TLR-4^25^. A summary of the mechanisms of action of HMGB1 in cardiac diseases, depending on subcellular location and redox state, is illustrated in Fig. 3.

The subcellular location of HMGB1 is a critical determinant of its function. Whilst the protective role of nuclear HMGB1 is increasingly recognised^42,49,50,96,117^, the specific intracellular functions of HMGB1 and the impact of post-translational modifications in cardiac diseases remain poorly understood. The available evidence suggests that maintaining nuclear HMGB1 is beneficial, with the inhibition of nuclear-to-cytoplasmic translocation reducing myocardial damage in heart failure^96^. Furthermore, reduced nuclear HMGB1 expression has been observed in murine pressure overload models and in human heart failure, particularly following the onset of decompensation, indicating that the loss of nuclear HMGB1 may contribute to disease progression^49^.

Physiologically, newly synthesised HMGB1 is predominantly transported into the nucleus, reinforcing the concept that upregulation of the nuclear pool is cardioprotective. However, under stress conditions, HMGB1 undergoes post-translational modifications, such as acetylation or oxidation, which facilitate its translocation to the cytoplasm^12,42^. Intracellular oxidation of frHMGB1 to dsHMGB1 promotes translocation from the nucleus to the cytoplasm^118^ and sustains autophagy through interaction with Beclin1^119^. Yet only a few studies in the context of cardiac disease have characterised the differential expression of HMGB1 in the nucleus versus the cytoplasm^49,96^. Given the importance of cytosolic HMGB1 in determining cell survival^119^, further research is required to elucidate the specific cytoplasmic functions and post-translational modifications of HMGB1 in cardiac pathology.

Genetic deletion of HMGB1 has also been shown to be harmful in both physiological and pathological states, whereas its overexpression is mostly protective. Global HMGB1 deletion (*Hmgb1*^−/−^)-induced lethal hypoglycemia in mice^120^, whilst mice with cardiomyocyte-specific conditional deletion (cTnI^Cre/+^−*Hmgb1*^fl/fl^) exhibited downregulated glucocorticoid receptor and PGC1-α signalling, leading to upregulation of glycolytic metabolism and impairment of heart development^121^. Conditional deletion of HMGB1 exacerbated CVB3-induced viral myocarditis^77^.

Conversely, overexpression of endogenous HMGB1 reduced DNA and myocardial damage in several disease models^48–50,97,117^. Whilst some studies reported that HMGB1 overexpression via viral vector transfection was deleterious^61,62,76,115^, this may have resulted from the use of non-native promoters that drive excessive protein expression, potentially leading to disrupted molecular interactions, mis-localisation, and cellular toxicity^122^. However, gene knock-in at the native locus ensures physiologically regulated expression, maintaining appropriate feedback mechanisms, post-translational modifications, and physiological subcellular location^122^, resulting in improved outcomes^48–50,97,117^.

A crucial aspect of HMGB1 biology is its redox state^25^. Although the majority of studies did not specify the redox state, it can be inferred based on receptor interactions and downstream signalling pathways. Extracellular frHMGB1 exclusively signals via CXCL12/CXCR4^18,22,23^, whereas dsHMGB1 binds TLR-2, TLR-4 and RAGE^18,25^. There is no overlap between frHMGB1 and dsHMGB1 signalling^25^. dsHMGB1 triggers a proinflammatory cascade, leading to the upregulation of proinflammatory cytokines^25^ and platelet activation^26^, which can exacerbate myocardial damage. Conversely, frHMGB1 promotes chemotaxis and repair^19,22^ (Fig. 3). frHMGB1 has also been reported to accelerate tissue repair of skeletal muscle, bone, and blood by transitioning tissue-resident stem cells from G_0_ to G_Alert_^19^.

Investigations into the effect of exogenous administration of rHMGB1 in IHD and HF yielded mixed results, likely dependent on the redox state of the preparation used. The only publication that clearly defined the redox state of the rHMGB1 reported that frHMGB1 signalling via CXCL12/CXCR4 was beneficial following MI^21^. The detrimental effects of rHMGB1 were largely associated with the activation of TLR-2, TLR-4, and RAGE signalling^37,59,64,82,88,89,101,116^, implicating dsHMGB1 as the primary mediator. Deletion of these receptors abolished the adverse effects of HMGB1, further supporting the notion that dsHMGB1 was used in these experiments^29,32–34,41,66,110,111^.

We attempted to clarify the redox state of the rHMGB1 when not stated in the publication by contacting the suppliers described in the materials (Tables 1–4). The majority responded that the redox status of their product was unknown. Only one supplier confirmed that one of their four preparations of the HMGB1 molecule was dsHMGB1 (1690-HMB, R&D), but the redox state of the remaining preparations could not be identified. Interestingly, four studies using HMGB1 from R&D Systems all reported beneficial effects in animals with acute MI or MI-induced HF^71,72,99,104^; however, none of these studies reported the product code or the redox state. The downstream signalling pathways reported included TLR-9^72^, TGF-β-Smad, ERK1/2, and HIF-1α/PI3K/AKT^71,99,104^, but none of them reported signalling via CXCL12-CXCR4, TLR-2, TLR-4 or RAGE^71,72,99,104^. Thus, we could not infer the redox state based on the information provided.

Other studies, despite not specifying the redox state, reported favourable outcomes following HMGB1 administration through upregulation of recruitment of bone marrow cells^48,105^, angiogenesis^45–47,69,72,98^, myocyte regeneration via c-kit^+^ stem cells^45–47,74,98^, and improvement in remodelling^98,99,104,105^ in MI and HF models. The contribution of c-kit^+^ cardiac stem cells to the repair of the adult mammalian heart has subsequently been largely discounted^75^. Interestingly, a non-oxidisable HMGB1 variant (3SHMGB1), engineered by substituting three conserved cysteine residues (C23, C45, and C106) with serine, exacerbated adverse remodelling and impaired cardiac function following MI, partly by abolishing angiogenic effects and promoting chemotaxis of cardiac fibroblasts via CXCR4, independent of CXCL12^21^. This contrasts with other tissues, including bone, blood, skeletal muscle, and liver, where the administration of 3SHMGB1 was beneficial^19,20^. These findings underscore the critical role of defining the ligand-receptor interaction as well as the redox state of HMGB1 when considering its effects on different tissues and cells.

None of the HMGB1 inhibition approaches selectively targeted either frHMGB1 or dsHMGB1. Glycyrrhizin binds to Box A or Box B, and dsHMGB1 had a higher binding affinity to glycyrrhizin compared to frHMGB1, resulting in a more stable glycyrrhizin–dsHMGB1 complex^123,124^. This could explain the beneficial effect of glycyrrhizin^38,40,59,64,65^. Monoclonal or polyclonal antibodies bind circulating HMGB1 without differentiating between the two redox forms^25^. Box A competitively inhibits HMGB1 by binding to TLR-4 and RAGE^125,126^, and also inhibits signalling via CXCL12/CXCR4^25^. Although the specific redox form of HMGB1 neutralised by antagonists remains unclear, dsHMGB1 may be predominantly affected in conditions characterised by chronic inflammation. Furthermore, the beneficial effect of inhibiting HMGB1 were largely associated with TLR-2/TLR-4/RAGE and their downstream signalling pathways^29,32–34,37,41,59,64,66,84,89,101,102,108,110,127–129^. However, non-selective inhibition of HMGB1 may also counteract the beneficial effects of frHMGB1, potentially worsening adverse remodelling^30,44^.

Modulation of HMGB1 in IHD^29,32,35,45–47,67,73,130^, HF^42,43,49,98,99,102,104^, myocarditis^76,77,86^, and chemotherapy-induced cardiomyopathy^111–117^ yielded conflicting results. However, in cardiomyopathies induced by diabetes^39,107–110^, trauma, stress^88,89^, or sepsis^82,84^, HMGB1 was consistently detrimental. In diabetic cardiomyopathy, chronic metabolic stress upregulates HMGB1, promoting inflammation and myocardial fibrosis. Although mice with cardiomyocyte-specific HMGB1 overexpression exhibited a reduced infarct size following MI^31^, mice with cardiomyocyte-specific HMGB1 overexpression with a streptozotocin-induced diabetic phenotype had larger infarcts and lower plasma HMGB1 levels compared to wild-type controls^131^. This suggests that the diabetic environment and trauma-, stress- or sepsis-induced cardiomyopathies may alter the functions of HMGB1, potentially by favouring the disulfide form.

The different effects of HMGB1 between disease states highlight the importance of distinct disease contexts and progression patterns. The available evidence supports the pivotal effect of the redox state of HMGB1. However, direct evidence is lacking due to the inability to differentiate frHMGB1 and dsHMGB1 in vivo and the failure to report the redox state of HMGB1. It is possible that in IHD, acute ischaemia results in cell necrosis, leading to passive release of frHMGB1 from the nucleus. However, prolonged ischaemia or excessive redox stress creates a microenvironment that is acidic due to anaerobic metabolism and lactate accumulation, which is likely to promote conversion of extracellular or intracellular frHMGB1 to dsHMGB1, leading to persistent inflammation, fibrosis, and adverse remodelling^25^.

HMGB1 is released actively under stress conditions and passively from damaged or necrotic cells. Circulating HMGB1 levels correlated positively with disease progression and severity in IHD, HF, and myocarditis^54–58,92,93^. Therapeutic interventions that improved cardiac health in both clinical and preclinical studies were associated with lower HMGB1 levels^79,80^ (Supplementary Data 1). However, HMGB1 is expressed in all nucleated cells, and thus, release is not limited to cardiac pathology, making it unsuitable for diagnostic purposes due to poor specificity as a result of its high sensitivity to comorbidities and injuries^93^ (Supplementary Note 3). Combining HMGB1 with other biomarkers (e.g. hsCRP, cTnI, NT-proBNP) may enhance its prognostic value^56,81,91–94^.

Despite the extensive literature, our understanding of the role of HMGB1 in cardiac pathologies remains incomplete. Firstly, some of the mechanisms of action described were not robustly verified. For example, HMGB1 was reported as promoting regeneration via resident c-kit^+^ cardiac stem cells^45,98,130^. However, the role of this mechanism has been questioned given the extremely limited contribution of these cells to cardiomyocyte renewal in adult mammals^75^. More sophisticated models, such as clonal expansion models using confetti mice^132^ or FUCCI (fluorescent ubiquitination-based cell cycle indicator) reporter mice^133^, are required to investigate this hypothesis further.

Second, distinguishing dsHMGB1 from frHMGB1, especially in vivo^18,25^, remains a significant challenge, limiting our understanding of the intracellular and extracellular oxidation dynamics. Redox-sensitive mass spectrometry approaches using differential cysteine alkylation could enable direct discrimination of HMGB1 redox isoforms in cardiac tissue and plasma. The development and validation of redox-state-specific HMGB1 antibodies or aptamers may provide an additional approach, although sensitivity and specificity could be limiting factors.

Finally, the timing and duration of HMGB1 release during cardiac events are still poorly defined. The temporal dynamics of HMGB1 release can determine whether it exerts protective or detrimental effects^42^, emphasising the importance of characterising how and when HMGB1 is released, and the redox state during different phases of cardiac injury. High-resolution time-course analyses of circulating and myocardial HMGB1 levels following injury may help define phase-specific functions. In parallel, cell-specific HMGB1 reporter or conditional knockout models could clarify the relative contribution of cardiomyocyte versus immune-derived HMGB1 over time, whilst single-cell and spatial transcriptomic approaches may provide complementary insight into the temporal and spatial context of HMGB1 signalling.

In conclusion, this review highlights the importance of HMGB1 in cardiac disorders. Its role is determined by its location and redox state. Intracellular HMGB1, particularly the nuclear fraction, is largely protective. However, its role following translocation to the cytoplasm and the effect of post-translational modification remain unclear. Cytoplasmic dsHMGB1 appears detrimental by facilitating nuclear export, thereby depleting the protective nuclear pool. Extracellularly, HMGB1 effects are dependent on its redox state: frHMGB1 exerts beneficial effects via the CXCL12–CXCR4 axis, whereas dsHMGB1 acts as a proinflammatory mediator through TLR-2, TLR-4, and RAGE signalling. To reconcile these apparently conflicting findings, more rigorous reporting of HMGB1 redox state and subcellular location is required. In addition, there is a pressing need to develop reliable methods for distinguishing between fully reduced HMGB1 (frHMGB1) and disulfide HMGB1 (dsHMGB1) in both tissue samples and circulation. These advances will be essential to realise the translational potential of targeting HMGB1-associated pathways for therapeutic benefit in cardiac disease.

## Methods

### Protocol and registration

This scoping review followed the Joanna Briggs Institute (JBI) methodology^134^ and was registered with the Open Science Framework (OSF) under their Scoping Review Protocol Guidance (https://osf.io/ajg9d/files/osfstorage/675575878fb4f95587563d2b). Results were reported according to the PRISMA extension for scoping reviews, ensuring structured reporting, transparency, and reproducibility (Supplementary Note 1)^135^.

### Eligibility criteria

Primary research studies investigating the role of HMGB1 in heart diseases were included. The exclusion criteria were as follows:

- Non-primary research, including correspondence, letters to the editor, editorials, book chapters, reviews, and conference abstracts.
- Studies that do not address HMGB1 as a primary or secondary focus.
- Studies without full-text availability or retracted.
- Studies unrelated to heart diseases, including those focused on cerebrovascular conditions, vascular diseases, or cardiac surgery.

The inclusion and exclusion criteria were confirmed by both primary reviewers before the formal screening process. Any disagreements regarding eligibility were resolved through discussion or consultation with a senior reviewer.

### Information sources

The search strategy encompassed three databases: *Embase (via Ovid), Medline (via Ovid), and Web of Science Core Collection*, searched separately without restrictions on date or language. The search terms included “HMGB1” and “heart diseases.” The strategy was developed collaboratively by the lead author (S.H.M.) and an academic librarian (K.S.). A full electronic search strategy was provided in Supplementary Note 2. The search was conducted on 8 October 2024. Citation management and deduplication were handled using EndNote and Covidence, and screening was performed in Rayyan.

### Selection of sources of evidence

Two independent reviewers evaluated all retrieved citations based on the pre-specified eligibility criteria using Rayyan. Full-text reviews were conducted for citations that meet the inclusion criteria or for undecidable abstracts. Reasons for exclusion at the full-text stage were documented. Conflicts were resolved through discussion or consultation with a senior reviewer. The outcomes of the search and study selection process were detailed in the scoping review and presented in a PRISMA flow diagram (Fig. 2).

### Data charting process

Data were extracted independently by two reviewers, focusing on study characteristics, outcomes, key findings, and proposed mechanisms relevant to the scoping review questions related to HMGB1 and heart diseases. A pilot extraction of ~10 articles was conducted by the lead author to refine the extraction tool. Extracted data were stored in Rayyan or Excel. Discrepancies between reviewers were documented and resolved through discussion or consultation with a senior reviewer. Adjustments were recorded in the final report to ensure transparency and consistency in the data extraction process.

### Synthesis of results

The results were presented using a PRISMA flow diagram. Data were summarised in graphical, diagrammatic, or tabulated formats, depending on the characteristics of the data. A narrative summary accompanied these visualisations, describing how the results align with the study objectives.

## Supplementary information

Supplementary Information

Supplementary Data 1

## Data Availability

All relevant data extracted and analysed during this study are included in this published article (and its supplementary information and data files). Additional raw extraction sheets used during the scoping process are available from the corresponding author upon reasonable request.
