# Supplementary material for: Scoping review of preclinical and clinical studies on the role of HMGB1 in heart disease
Source: NPJ Cardiovasc Health. 2026 Apr 9;3:20. doi: 10.1038/s44325-026-00119-4 (PMC13066481; doi:10.1038/s44325-026-00119-4)
Supplement: Supplementary file 1 — Supplementary Information [file 44325_2026_119_MOESM1_ESM.pdf]

Supplementary Note 1. PRISMA-ScR Checklist

Preferred Reporting Items for Systematic reviews and Meta-Analyses extension for Scoping Reviews (PRISMA-ScR) Checklist

SECTION	ITEM	PRISMA-ScR CHECKLIST ITEM	REPORTED ON PAGE #
**TITLE**			
Title	1	Identify the report as a scoping review.	1
**ABSTRACT**			
Structured summary	2	Provide a structured summary that includes (as applicable): background, objectives, eligibility criteria, sources of evidence, charting methods, results, and conclusions that relate to the review questions and objectives.	2
**INTRODUCTION**			
Rationale	3	Describe the rationale for the review in the context of what is already known. Explain why the review questions/objectives lend themselves to a scoping review approach.	4-5
Objectives	4	Provide an explicit statement of the questions and objectives being addressed with reference to their key elements (e.g., population or participants, concepts, and context) or other relevant key elements used to conceptualize the review questions and/or objectives.	4-5
**METHODS**			
Protocol and registration	5	Indicate whether a review protocol exists; state if and where it can be accessed (e.g., a Web address); and if available, provide registration information, including the registration number.	36
Eligibility criteria	6	Specify characteristics of the sources of evidence used as eligibility criteria (e.g., years considered, language, and publication status), and provide a rationale.	36
Information	7	Describe all information sources in the	37

SECTION	ITEM	PRISMA-ScR CHECKLIST ITEM	REPORTED ON PAGE #
sources*		search (e.g., databases with dates of coverage and contact with authors to identify additional sources), as well as the date the most recent search was executed.	
Search	8	Present the full electronic search strategy for at least 1 database, including any limits used, such that it could be repeated.	37
Selection of sources of evidence†	9	State the process for selecting sources of evidence (i.e., screening and eligibility) included in the scoping review.	37
Data charting process‡	10	Describe the methods of charting data from the included sources of evidence (e.g., calibrated forms or forms that have been tested by the team before their use, and whether data charting was done independently or in duplicate) and any processes for obtaining and confirming data from investigators.	37
Data items	11	List and define all variables for which data were sought and any assumptions and simplifications made.	37
Critical appraisal of individual sources of evidence§	12	If done, provide a rationale for conducting a critical appraisal of included sources of evidence; describe the methods used and how this information was used in any data synthesis (if appropriate).	n/a
Synthesis of results	13	Describe the methods of handling and summarizing the data that were charted.	38
**RESULTS**			
Selection of sources of evidence	14	Give numbers of sources of evidence screened, assessed for eligibility, and included in the review, with reasons for exclusions at each stage, ideally using a flow diagram.	6
Characteristics of sources of	15	For each source of evidence, present characteristics for which data were charted	6

SECTION	ITEM	PRISMA-ScR CHECKLIST ITEM	REPORTED ON PAGE #
evidence		and provide the citations.	
Critical appraisal within sources of evidence	16	If done, present data on critical appraisal of included sources of evidence (see item 12).	n/a
Results of individual sources of evidence	17	For each included source of evidence, present the relevant data that were charted that relate to the review questions and objectives.	6-30
Synthesis of results	18	Summarize and/or present the charting results as they relate to the review questions and objectives.	6-30
**DISCUSSION**			
Summary of evidence	19	Summarize the main results (including an overview of concepts, themes, and types of evidence available), link to the review questions and objectives, and consider the relevance to key groups.	30-34
Limitations	20	Discuss the limitations of the scoping review process.	34-35
Conclusions	21	Provide a general interpretation of the results with respect to the review questions and objectives, as well as potential implications and/or next steps.	35-36
**FUNDING**			
Funding	22	Describe sources of funding for the included sources of evidence, as well as sources of funding for the scoping review. Describe the role of the funders of the scoping review.	38

JBI = Joanna Briggs Institute; PRISMA-ScR = Preferred Reporting Items for Systematic reviews and Meta-Analyses extension for Scoping Reviews.

* Where *sources of evidence* (see second footnote) are compiled from, such as bibliographic databases, social media platforms, and Web sites.

† A more inclusive/heterogeneous term used to account for the different types of evidence or data sources (e.g., quantitative and/or qualitative research, expert opinion, and policy documents) that may be eligible in a scoping review as opposed to only studies. This is not to be confused with *information sources* (see first footnote).

‡ The frameworks by Arksey and O'Malley (6) and Levac and colleagues (7) and the JBI guidance (4, 5) refer to the process of data extraction in a scoping review as data charting.

§ The process of systematically examining research evidence to assess its validity, results, and relevance before using it to inform a decision. This term is used for items 12 and 19 instead of "risk of bias" (which is more applicable to systematic reviews of interventions) to include and acknowledge the various sources of evidence that may be used in a scoping review (e.g., quantitative and/or qualitative research, expert opinion, and policy document).

From: Tricco AC, Lillie E, Zarin W, O'Brien KK, Colquhoun H, Levac D, et al. PRISMA Extension for Scoping Reviews (PRISMAScR): Checklist and Explanation. Ann Intern Med. 2018;169:467–473. doi: 10.7326/M18-0850.

Supplementary Note 2. Search Strategies

Embase via Ovid

Source: Embase 1974 to present
Run: 8 October 2024

1 high mobility group B1 protein/
2 high mobility group B1*.tw,kf.
3 HMGB1*.tw,kf.
4 HMGB-1*.tw,kf.
5 High mobility group box 1*.tw,kf.
6 high mobility group 1 protein*.tw,kf.
7 high mobility group box protein 1.tw,kf.
8 HMG-1.tw,kf.
9 HMG1.tw,kf.
10 Amphoterin.tw,kf.
11 or/1-10
12 exp cardiovascular disease/
13 (heart disease* or cardiac disease* or cardiovascular disease* or cardio-vascular disease*).tw,kf.
14 (cardiovascular risk* or cardio-vascular risk*).tw,kf.
15 (atherosclero* or athero-sclero*).tw,kf.
16 coronary artery disease*.tw,kf.
17 (myocardial infarct* or heart infarct* or heart attack* or cardiac infarct*).tw,kf.
18 (heart failure* or myocardial failure* or cardiac failure*).tw,kf.
19 (cardiomyopath* or cardio-myopath* or cardiac myopath*).tw,kf.
20 myocardial ischemia reperfusion injury/
21 (myocardial ischemia reperfusion injur* or myocardial ischaemia reperfusion injur*).tw,kf.
22 myocardial I/R-induced injur*.tw,kf.
23 myocardial I/R* injur*.tw,kf.
24 cardiac hypertrophy.tw,kf.
25 (isch?emi* reperfusion injur* and heart*).tw,kf.
26 heart infarction/
27 (cardiac regeneration or heart regeneration or cardiovascular regeneration or cardio-vascular regeneration).tw,kf.

28 (cardiac function* or heart function* or cardiovascular function* or cardio-vascular function*).tw,kf.
29 (cardiac protect* or cardioprotect* or cardio-protect* or heart protect* or cardiovascular protect* or cardio-vascular protect*).tw,kf.
30 or/12-29
31 11 and 30
32 limit 31 to "review"
33 31 not 32

Medline via Ovid

Source: Medline (Ovid MEDLINE® Epub Ahead of Print, In-Process & Other Non-Indexed Citations, Ovid MEDLINE® Daily and Ovid MEDLINE®) 1946 to present
Run: 8 October 2024

1 HMGB1 Protein/
2 high mobility group B1*.tw,kf.
3 HMGB1*.tw,kf.
4 HMGB-1*.tw,kf.
5 High mobility group box 1*.tw,kf.
6 high mobility group 1 protein*.tw,kf.
7 high mobility group box protein 1.tw,kf.
8 HMG-1.tw,kf.
9 HMG1.tw,kf.
10 Amphoterin.tw,kf.
11 or/1-10
12 exp Cardiovascular Diseases/
13 (heart disease* or cardiac disease* or cardiovascular disease* or cardio-vascular disease*).tw,kf.
14 (cardiovascular risk* or cardio-vascular risk*).tw,kf.
15 (atherosclero* or athero-sclero*).tw,kf.
16 coronary artery disease*.tw,kf.
17 (myocardial infarct* or heart infarct* or heart attack* or cardiac infarct*).tw,kf.

18 (heart failure* or myocardial failure* or cardiac failure*).tw,kf.
19 (cardiomyopath* or cardio-myopath* or cardiac myopath*).tw,kf.
20 (myocardial ischemia reperfusion injur* or myocardial ischaemia reperfusion injur*).tw,kf.
21 myocardial I/R-induced injur*.tw,kf.
22 myocardial I/R* injur*.tw,kf.
23 cardiac hypertrophy.tw,kf.
24 (isch?emi* reperfusion injur* and heart*).tw,kf.
25 (cardiac regeneration or heart regeneration or cardiovascular regeneration or cardio-vascular regeneration).tw,kf.
26 (cardiac function* or heart function* or cardiovascular function* or cardio-vascular function*).tw,kf.
27 (cardiac protect* or cardioprotect* or cardio-protect* or heart protect* or cardiovascular protect* or cardio-vascular protect*).tw,kf.
28 or/12-27
29 11 and 28
30 limit 29 to ("review" or "systematic review")
31 29 not 30

Web of Science Core Collection

Source: Web of Science Core Collection, all editions
Editions included: Science Citation Index Expanded (SCI-EXPANDED) 1900-present
Social Sciences Citation Index (SSCI) 1900-present
Arts & Humanities Citation Index (AHCI) 1975-present
Conference Proceedings Citation Index (CPCS-S) - Science 1990-present
Conference Proceedings Citation Index (CPCI-SSH) - Social Sciences & Humanities 1990-present
Book Citation Index (BKCI-S) - Science 2005-present
Book Citation Index (BKCI-SSH) - Social Sciences & Humanities 2005-present

Emerging Sources Citation Index (ESCI) 2015-present
Current Chemical Reactions (CCR-EXPANDED) 1985-present
Index Chemicus (IC) 1993-present
Run: 8 October 2024

1: TS=("high mobility group B1*")
2: TS=(HMGB1*)
3: TS=(HMGB-1*)
4: TS=("High mobility group box 1*")
5: TS=(high mobility group 1 protein*)
6: TS=("high mobility group box protein 1")
7: TS=(HMG-1 or HMG1)
8: TS=(Amphoterin)
9: #1 OR #2 OR #3 OR #4 OR #5 OR #6 OR #7 OR #8
10: TS=("heart disease*" or "cardiac disease*" or "cardiovascular disease*" or "cardio-vascular disease*")
11: TS=("cardiovascular risk*" or "cardio-vascular risk*")
12: TS=(atherosclero* or athero-sclero*)
13: TS=("coronary artery disease*")
14: TS=("myocardial infarct*" or "heart infarct*" or "heart attack*" or "cardiac infarct*")
15: TS=("heart failure*" or "myocardial failure*" or "cardiac failure*")
16: TS=(cardiomyopath* or cardio-myopath*)
17: TS=(cardiac myopath*)
18: TS=("myocardial ischemia reperfusion injur*" or "myocardial ischaemia reperfusion injur*")
19: TS=("myocardial I/R-induced injur*")
20: TS=("myocardial I/R* injur*")
21: TS=("cardiac hypertrophy")
22: TS=("ischemi* reperfusion injur*" and heart*)
23: TS=("ischaemi* reperfusion injur*" and heart*)
24: TS=("cardiac regeneration" or "heart regeneration" or "cardiovascular regeneration" or "cardio-vascular regeneration")
25: TS=("cardiac function*" or "heart function*" or "cardiovascular function*" or "cardio-vascular function*")
26: TS=("cardiac protect*" or cardioprotect* or cardio-protect*)
27: TS=("heart protect*")
28: TS=("cardiovascular protect*" or "cardio-vascular protect*")

29: #10 OR #11 OR #12 OR #13 OR #14 OR #15 OR #16 OR #17 OR #18 OR #19 OR #20 OR #21 OR #22 OR #23 OR #24 OR #25 OR #26 OR #27 OR #28
30: #29 AND #9
31: #29 AND #9 and Review Article (Exclude – Document Types)

Supplementary Note 3. HMBG1 as a Clinical Prognostic Biomarker in Ischaemic Heart Diseases

Recent clinical studies have evaluated the association between circulating HMGB1 levels and the severity and progression of coronary artery disease (CAD). These investigations, encompass acute coronary syndromes (ACS) such as ST-elevation myocardial infarction (STEMI), non-STEMI (NSTEMI), and unstable angina, highlight the potential of HMGB1 as a prognostic biomarker.

HMGB1 levels reflect both the severity of cardiac injury and functional status following acute coronary syndrome (ACS).

Patients with CAD had elevated HMGB1 levels compared to healthy individuals[1-3]. Moreover, individuals with acute myocardial infarction (AMI) had higher circulating HMGB1 than those with unstable or stable angina, and HMGB1 levels correlated with high-sensitivity C-reactive protein (hs-CRP) and cardiac troponin I (cTnI)[2]. Circulating HMGB1 also correlated positively with infarct size and inversely with residual left ventricular ejection fraction (LVEF) in both STEMI and NSTEMI patients[4]. Specifically, cut-off values of 6.2 ng/mL for STEMI and 5.9 ng/mL for NSTEMI predicted infarct extending transmurally by >75%, while HMGB1 thresholds of 7.2 ng/mL for STEMI and 6.4 ng/mL for NSTEMI correlated with reduced LVEF at six months post-ACS[4].

HMGB1 can predict major cardiovascular events and mortality following ACS.

Coronary lesion length, Gensini score, LVEF, IL-6 and HMGB1 demonstrated strong predictive value in a comparative analysis of patients with and without major adverse cardiovascular events (MACE)[5]. Combined parameters of HMGB1 and IL-6 yielded an AUC of 0.922, with a sensitivity of 90.62% and a specificity of 82.95%, which is comparable to the Gensini score (AUC 0.941, sensitivity 81.25%, specificity 93.18%).

These findings suggest that HMGB1 in combination with IL-6 may serve have comparable predictive value to MACE [5]. The risk of developing an acute cardiac event within one month post-discharge from ICU was significantly higher in patients with HMGB1 levels >0.6 μg/L on admission[6]. Additionally, elevated levels of NT-proBNP and HMGB1 were associated with a higher incidence of cerebral infarction following AMI[7].

HMGB1 levels are correlated with mortality following ACS.

HMGB1 levels were markedly higher in patients who succumbed to STEMI compared to survivors and healthy individuals[8]. In addition, research involving 258 patients with unstable angina/NSTEMI demonstrated that elevated HMGB1 levels at admission were associated with an increased rate of cardiovascular mortality. Multivariate analysis confirmed HMGB1 as an independent prognostic marker of mortality, and combination with biomarkers such as hsCRP, cTnI and BNP further improved early risk stratification in UA/NSTEMI patients[9]. Moreover, in a study of 302 consecutive patients undergoing percutaneous coronary intervention, elevated preoperative HMGB1 predicted post-procedure myocardial injury defined by peak values of CK-MB and cTnI, and was associated with the worst event-free survival over a one-year follow-up[10].

Circulating HMGB1 levels are associated with worse cardiopulmonary function.

HMGB1 levels were significantly higher in patients recovering from AMI than in age- and body mass index-matched healthy controls, and inversely correlated with both cardiopulmonary and cardiac performance as measured by exercise testing, Doppler echocardiography and autonomous function[11,12]. These findings suggest that HMGB1 levels are associated with functional impairment and may remain elevated even after

recovery from acute injury.

Elevated circulating HMGB1 may predict vessel stenosis and plaque characteristics.

HMGB1 and hsCRP levels were significantly elevated in patients with unstable angina compared to those with stable angina[13]. Additionally, both HMGB1 and TNF-α levels were elevated in patients with ACS[14]. A positive correlation was observed between HMGB1 levels and the Gensini score, as assessed by coronary angiography, in patients with stable angina[13] and in those with ACS[14]. In ACS patients, HMGB1 levels also showed a positive association with the number of coronary artery lesions and an inverse correlation with LVEF[14]. These findings suggest that HMGB1, TNF-α, and hsCRP may serve as useful biomarkers for assessing the severity of coronary artery lesions in CAD. Furthermore, both hsTnT and HMGB1 were identified as independent predictors of the non-calcified plaque burden, which refers to the extent of atherosclerotic plaques lacking significant calcium deposits and often considered vulnerable to rupture. By combining hsTnT and HMGB1, a high positive predictive value for the presence of non-calcified and remodeled plaques (96% and 77%, respectively) was achieved in patients[15].

Elevated levels of HMGB1 are associated with the development of CAD.

In patients aged ≤50 years undergoing angiography for chest pain, HMGB1 levels were significantly higher in those diagnosed with CAD compared to individuals without angiographic evidence of plaque[16]. In a study of 476 non-diabetic patients undergoing coronary angiography for suspected or known CAD, serum HMGB1 levels were positively correlated with a 10-year risk of CAD as assessed by the Framingham Risk Score, independent of undiagnosed abnormal glucose regulation[17].

HMGB1 levels are higher in patients with NSTEMI and STEMI compared to those with unstable or stable angina, and correlated with IL-17A and hsCRP. However, only IL-17A was identified as an independent predictor for CAD on regression analysis encompassing both angina and acute myocardial infarction, suggesting that the HMGB1/IL-17A axis may play a role in the pathogenesis of atherosclerotic CAD[18].

Bioinformatic analysis of gene expression data from patients with CAD and healthy controls identified *HMGB1*, along with *TLR3*, *MLKL* and *NDRG2*, as significantly upregulated genes associated with necroptosis. The authors suggested that these genes may serve as prospective biomarkers for CHD diagnosis and implicated necroptosis in CHD progression through inflammatory pathways[19].

Platelets play a crucial role in the pathogenesis of coronary artery disease, as dsHMGB1 has been demonstrated to induce coagulation[20].

HMGB1 is highly expressed in platelets isolated from affected coronary arteries compared to those from peripheral blood, and it promoted a procoagulant endothelial phenotype[21]. However, the prognostic utility of HMGB1 from platelets remains uncertain, as no significant differences were observed in HMGB1 expression on the platelets of patients with symptomatic CAD[22].

HMGB1 levels have been linked to the presence of comorbidities and gender differences. In patients with acute myocardial infarction, HMGB1 levels correlated with cardiac-specific biomarkers (NT-proBNP and cTnI), independent of heart failure. There was also a positive association with co-morbidities, including hypertension, diabetes, prior heart disease and reduced LVEF[23]. Moreover, among CAD patients,

those with concurrent inflammatory rheumatic diseases had higher cytoplasmic HMGB1 and lower total cardiac HMGB1, suggesting that these conditions may promote HMGB1 translocation and extracellular release[24]. Additionally, sex differences in monocyte transcriptomes have been observed; female patients display a stronger migratory signature, whereas IL-4, INS, and HMGB1 signalling pathways are more pronounced in males[25].

Conflicting reports of the utility of HMGB1 as a biomarker

In patients with CAD complicated by carotid atherosclerosis, combination therapy with amlodipine and atorvastatin significantly reduced intima-media thickness, CK-MB and LDH levels and improved lipid profiles compared to atorvastatin monotherapy. Additionally, patients receiving combination therapy exhibited significantly lower HMGB1 levels than those treated with atorvastatin alone [26]. Similarly, preloading patients with stable angina pectoris with atorvastatin prior to PCI significantly decreased peripheral expression of TLR-2 and TLR-4, as well as of HMGB1 and cardiac biomarkers (cTnI and CK-MB)[27]. CAD patients treated with statins had lower miR-218 levels, which negatively correlated with PCSK9, HMGB1 and TLR-4 in a dose-dependent manner. However, the study did not report clinical outcome data[28]. Conversely, another study showed that HMGB1 levels increased significantly following atorvastatin administration in CAD patients with type-2 diabetes mellitus[29]. These inconsistencies between HMGB1 levels and clinical outcomes, cast doubt on its reliability as a prognostic biomarker for therapeutic response.

Taken together, the available evidence supports the prognostic value of HMGB1 in CAD patients.

Elevated HMGB1 levels are associated with greater disease severity, increased

myocardial injury and worse clinical outcomes in ACS. Additionally, HMGB1 may predict angiographic findings and disease progression, although further research is needed to clarify its relationship with comorbidities and interventional responses.

Supplementary Note 4. Other Cardiomyopathies

Congenital/Dilated Cardiomyopathy

HMGB1 as a Prognostic Biomarker

A study of 67 patients with dilated cardiomyopathy (DCM)demonstrated reported elevated HMGB1 and NT-proBNP levels were associated with increased all-cause mortality. Multivariable analysis showed that HMGB1 was an independent risk factor for mortality in DCM, regardless of NT-proBNP levels, age or gender[30]. Similarly, in a study involving 84 neonatal patients with congenital heart disease (CHD), plasma levels of NLRP3 and HMGB1 were significantly higher in deceased patients compared to survivors. Elevated levels of these markers correlated with significantly lower two-year survival[31]. These findings suggest that HMGB1 could serve as a potential prognostic biomarker for both CHD and DCM.

However, the prognostic utility of HMGB1 may not extend to pediatric patients with CHD-associated pulmonary arterial hypertension (CHD-PAH). In pediatric CHD-PAH patients, neither HMGB1 nor NT-proBNP levels differed significantly between patients and controls, and did not correlate with severity of pulmonary hypertension. Therefore. unlike in adults[32], HMGB1 does not appear to be a reliable biomarker for PAH in pediatric CHD[33].

Collectively, HMGB1 may serve as a potential prognostic marker in adult DCM and in some populations of paediatric CHE.

Conflicting Effects of Exogenous Administration of HMGB1

Exogenous administration of HMGB1 has demonstrated beneficial was in δ-sarcoglycan-deficient hamsters, a model of DCM. Treatment with HMGB1 fragments preserved cardiac function and improved survival compared to untreated controls. This protective effect was associated with reduced fibrosis, oxidative stress and macrophage infiltration, as well as enhanced angiogenesis.

Additionally, treated animals exhibited more organized mitochondrial cristae. Immunohistochemical analysis revealed an increased presence of PDGFRα$^+$ and CD106$^+$ cells, and a higher density of CXCR4$^+$ cells within CXCL12$^+$ cell-populated regions in the myocardium. The authors concluded that systemic administration of a Box-A HMGB1 fragment mitigated adverse remodeling in DCM by facilitating the recruitment of bone marrow-derived mesenchymal stem cells to the injured myocardium[34].

Conversely, HMGB1 has been implicated in deleterious inflammatory signalling in a Duchenne Muscular Dystrophy (DMD) cardiomyopathy model[35]. Mdx animals (C57BL6/10ScSn-DMDmdx/J) developed progressive fibrosis of the myocardial tissue together with persistent inflammation and complement activation. Pentraxin 3 (PTX3), a key component of the innate immune system, plays a role in complement pathway activation, inflammation and the coordination of macrophage and dendritic cell functions in regulating apoptosis and necrosis. PTX3 expression levels in mdx myocardium was age-dependent and correlated with levels of inflammatory markers (TLR-2/4/5/9, IL-1R, and MyD88) and alarmins (IL-33, HMGB1, and S100β). Treatment with an inflammatory 5regulator (ONX-0914) attenuated the inflammation, with associated reduction in HMGB1[35].

These findings suggest that exogenous administration of a Box-A fragment of HMGB1 facilitates the recruitment of bone marrow-derived stem cells to promote myocardial repair, whereas HMGB1 may also mediate inflammation that exacerbates cardiomyopathy.

Metabolism-Associated Cardiomyopathy

Detrimental Role of HMGB1 in Preclinical Models

Obesity and metabolic dysregulation have been identified as increasing risk factors for cardiomyopathy[36,37]. Elevated levels of saturated fatty acid are commonly observed in patients with diabetes, obesity and other metabolic disorders. In cardiomyocytes treated with palmitic acid, upregulation of the long noncoding RNA (lncNRA) metastasis-associated lung adenocarcinoma

transcript 1 (MALAT1) was linked to reduced cell viability and increased expression of HMGB1, TLR-4, and NF-κB. This activation of the HMGB1/TLR4/NF-κB signaling pathway promoted inflammation, suggesting a regulatory role of lncRNA MALAT1/HMGB1 in lipid-induced cardiomyocyte injury[36].

Dietary patterns also influence HMGB1 levels. Mice fed a Western diet exhibited elevated serum free fatty acid levels and increased activation of the HMGB1/TLR-4/TRAF6 axis, indicating a potential role for HMGB1 in Western diet-induced inflammation[38].

Adipose tissue is an endocrine and paracrine organ that secretes adipokines, including HMGB1, visfatin, RBP-4, and TNF[37]. Conditioned media from visceral or subcutaneous adipocytes of obese mice impaired the differentiation of cardiac precursor cells and induced apoptosis in cardiomyocytes in the presence of adipokines. Anti-HMGB1 antibody reversed these effects, suggesting that HMGB1 plays a significant role in impairing cardiac differentiation and cell survival[37].

The available evidence suggests HMGB1 mediates inflammation as a result of free saturated fatty acids and products from adipocytes from obese mice, leading to deleterious effects on cardiomyocytes.

HMGB1 and Lipotoxicity in Clinical Studies

Lipotoxicity can contribute to myocardial injury and dysfunction, with HMGB1 potentially mediates this process[39,40].

Epicardial adipose tissue thickness was found to correlate with Lipid Accumulation Product index in patients with coronary artery disease (CAD) undergoing coronary artery bypass grafting (CABG). This correlation was associated with increased expression of RAGE, HMGB1, TLR-4 and MyD88 in epicardial adipose tissue, suggesting that the axis may be involved in inflammation and metabolic myocardial dysfunction induced by prolonged

lipotoxicity[40].

Obese pediatric patients had higher HMGB1 levels compared to healthy controls. HMGB1 levels correlated positively with total and LDL cholesterol levels but did not show a significant association with echocardiographic parameters, indicating a potential link between HMGB1 and lipid metabolism without early structural cardiac alterations[39].

Collectively, these studies indicate that HMGB1 expression is associated with circulating lipids, and may result in myocardial injury secondary to inflammation. However, the number of the studies is limited.

Age-Associated Cardiomyopathy is Mediated by HMGB1

HMGB1 has been shown to be associated with ageing-associated cardiomyopathy by modulating inflammatory responses and redox stress[41,42].

In the SAMP8 murine model of accelerated ageing, upregulation of HMGB1 was accompanied by increased expression of TLR-2,TLR-4, NF-κB p65, IL-1β, IL-6, TNF, COX-2, IFN-γ and M1-like $CD68^{+}$ macrophages in the heart. Conversely, the expression of M2 $CD36^{+}$ macrophages and IL-10 was downregulated. These findings suggest that HMGB1–TLR-2/TLR-4 signalling promotes M1 macrophage polarization, potentially exacerbating cardiac dysfunction in ageing hearts [41].

Adiponectin, an adipocyte-derived hormone with anti-inflammatory and antioxidant properties in cardiovascular disease, has been implicated in counteracting HMGB1-mediated oxidative stress[42]. Adiponectin expression was downregulated in cardiomyocyte senescence induced by D-galactose. Overexpression of adiponectin inhibited oxidative stress, which was associated with the downregulation of HMGB1, whereas HMGB1 overexpression abolished the effect[42]. These findings suggest that adiponectin may exert a protective effect against age-related cardiac dysfunction by inhibiting the activity of HMGB1.

Taken together, HMGB1 has been shown to mediate pathogenesis of age-

associated cardiomyopathy.

Arrhythmias

HMGB1 influences cardiomyocyte contractility and ion channel function and also activates proinflammatory pathways, all of which contribute to cardiac rhythm disturbances.

Ion Channels and Contractility

HMGB1 was found to enhance sarcoplasmic reticulum Ca^{2+} leak in cardiomyocytes through Ca^{2+} sparks, impairing excitation–contraction coupling. This effect was mediated by the TLR-4–ROS signaling pathway; inhibition of TLR-4 or ROS prevented HMGB1-induced disruption of cardiac excitation–contraction coupling[43]. Furthermore, HMGB1 treatment for 24 h reduced current densities of Kv4.3 and Kv4.2 channels in cardiomyocytes in a dose-dependent manner, slightly prolonging action potential duration. Treatment with soluble RAGE, which blocks ligand binding to cell-surface RAGE, partially restored cardiac transient outward potassium current (Ito) density and Kv4 protein expression, suggesting that HMGB1 downregulates Ito currents, at least partially, via HMGB1–RAGE interaction[44].

HMGB1 can also impair cardiac contractility. Treatment of isolated cardiomyocytes with rHMGB1 led to 70% reduction in sarcomere shortening and a 50% decrease in peak Ca^{2+} transients within five minutes. The immediate negative inotropic effects of HMGB1 on cardiac contractility and calcium homeostasis were partially reversible upon washout. HMGB1 induced PKC-ε translocation, and inhibition of PKC significantly attenuated these negative effects, suggesting that HMGB1 disrupts sarcomere function by modulating membrane calcium influx[45].

MI-Induced Arrhythmia

MI can induce ventricular arrhythmias, in part mediated by HMGB1.
Selective ablation of the distal part of the ligament of Marshall suppressed ventricular arrhythmias in AMI patients, reduced markers of oxidative stress

(MDA, SOD), apoptosis (caspase-3) and proinflammatory cytokines (HMGB1, TNF, IL-6)[46].

Furthermore, MI triggered HMGB1 upregulation and translocation within the paraventricular nucleus, leading to sympathetic overactivation via the ERK1/2 signaling pathway. Bilateral PVN microinjection of an anti-HMGB1 polyclonal antibody downregulated expression of HMGB1 and p-ERK, significantly reduced baseline renal sympathetic nerve activity and inducible ventricular arrhythmias[47].

Atrial Fibrillation (AF)

Genetic Evidence

Genetic variations in HMGB1 have also been implicated in susceptibility to AF. *HMGB1*, along with other ferroptosis-related genes (*TGFBR1*, *CAV1*, and *CD44*), was upregulated in patients with valvular AF compared to those with valvular sinus rhythm[48]. Further, the 3'UTR variant rs1045411T/C may influence the risk of late-onset AF in the Chinese Han population[49] and HMGB1 rs2249825 polymorphism is associated with postoperative AF following CABG with cardiopulmonary bypass[50].

Clinical Prognostic Markers

Elevated HMGB1 levels were associated with the occurrence of AF and AF-associated thrombosis. Patients with persistent or paroxysmal AF exhibited higher HMGB1 levels compared to matched controls, along with increased hs-CRP, MDA and SOD. This suggests an association between HMGB1 levels and oxidative stress in the development of AF[51]. Furthermore, elevated levels of postoperative HMGB1 were associated with recurrence in patients with paroxysmal AF who underwent cryoballoon ablation[52]. AF patients who developed atrial thrombosis had higher HMGB1 levels in biopsies from the left atrial appendage, along with increased expression of MyD88, p-NFκB/NFκB and tissue factor, compared to AF patients without thrombosis and those in sinus rhythm[53].

Collectively, these findings indicate that HMGB1 is involved in arrhythmogenesis, especially in AF and post-MI ventricular arrhythmias. HMGB1 may regulate signal transduction from the central nervous system and peripheral inflammatory responses. Additionally, it influences ion channel function and calcium currents, disrupting excitation-contraction coupling and further impairing inotropic effects, which may exacerbate the onset and progression of arrhythmias. HMGB1 appears to contribute to arrhythmogenesis, least in part, through modulation of inflammation and activation of ion channels.

Cancer-Associated Cardiomyopathy

Upregulation of HMGB1 has been associated with cardiac injury and dysfunction secondary to adjuvant cancer therapies and cancer-related cachexia. Conversely, interventions that mitigate cardiac injury are associated with downregulation of HMGB1.

Animals treated with the combination of a PD-1 inhibitor and irradiation exhibited more extensive myocardial injury, fibrosis and immune infiltration, particularly of $CD8^+$ cells, compared to those receiving individual therapy or untreated controls. The severity of the injury correlated with expression of HMGB1 and NF-κB, along with elevated levels of proinflammatory markers, including IL-1β, IL-6 and TNF. These findings suggest that combined PD-1 inhibitor and irradiation therapy exacerbates myocardial injury, potentially through the upregulation of HMGB1[54].

Radiation of the cardiothoracic region resulted in left ventricular systolic dysfunction, myocardial fibrosis and cardiac injury, accompanied by elevated myocardial HMGB1 protein levels[55]. Administration of the traditional Chinese medicinal herb Danggui Buxue Tang (DBT) significantly reduced myocardial fibrosis and improved left ventricular systolic function. These protective effects were associated with upregulation of Nrf2 and downregulation of HMGB1 protein levels in the myocardium[55].

Cancer-induced cachexia impaired function of multiple organs, including the heart, although the underlying mechanisms remain unclear. Administration of rHMGB1 in cultured cardiomyocytes induced apoptosis and autophagy by activating the NF-κB and PI3K/AKT pathways via RAGE/TLR-4 signalling.

Myocardial injury was attenuated by the administration of berberine, a plant-derived immunomodulator, in a cachectic rat model. The cardioprotective effects of berberine were associated with the upregulation of miR-181c-5p and miR-340-5p and downregulation of the HMGB1/TLR-4/RAGE axis in cardiomyocytes[56].

The collective evidence suggests that HMGB1 may mediate the progression of myocardial injury and serve as a marker of injury severity in cancer and chemoradiotherapy-associated cardiomyopathies.

References

1 Benlier, N., Erdogan, M. B., Kecioglu, S., Orhan, N. & Cicek, H. Association of high mobility group box 1 protein with coronary artery disease. *Asian cardiovascular & thoracic annals* **27**, 251-255 (2019). https://doi.org/10.1177/0218492319835725

2 Yao, H. C. *et al.* Correlation between serum high-mobility group box-1 levels and high-sensitivity C-reactive protein and troponin I in patients with coronary artery disease. *EXPERIMENTAL AND THERAPEUTIC MEDICINE* **6**, 121-124 (2013). https://doi.org/10.3892/etm.2013.1095

3 Yan, X. X. *et al.* Increased serum HMGB1 level is associated with coronary artery disease in nondiabetic and type 2 diabetic patients. *ATHEROSCLEROSIS* **205**, 544-548 (2009). https://doi.org/10.1016/j.atherosclerosis.2008.12.016

4 Andrassy, M. *et al.* HMGB1 as a predictor of infarct transmurality and functional recovery in patients with myocardial infarction. *JOURNAL OF INTERNAL MEDICINE* **270**, 245-253 (2011). https://doi.org/10.1111/j.1365-2796.2011.02369.x

5 He, W. *et al.* IL-6 and HMGB1 Levels for Predicting Major Adverse Vascular Events after Percutaneous Coronary Intervention in Patients with Acute Coronary Syndrome. *Heart Surgery Forum* **27**, E960-E967 (2024). https://doi.org/10.59958/hsf.7497

6 Yamada, S., Yakabe, K., Hil, J. I., Lmaizumi, H. & Maruyama, I. New high mobility group box 1 assay system. *CLINICA CHIMICA ACTA* **372**, 173-178 (2006). https://doi.org/10.1016/j.cca.2006.04.016

7 Chen, J., Zhang, W., Wu, Y. Q., Chen, H. & Zhao, J. F. Correlations of acute myocardial infarction complicated by cerebral infarction with insulin resistance, adiponectin and HMGB1. *EUROPEAN REVIEW FOR MEDICAL AND PHARMACOLOGICAL SCIENCES* **23**, 4425-4431 (2019).

8 Sorensen, M. V., Pedersen, S., Mogelvang, R., Skov-Jensen, J. & Flyvbjerg, A. Plasma High-Mobility Group Box 1 Levels Predict Mortality After ST-Segment Elevation Myocardial Infarction. *JACC-CARDIOVASCULAR INTERVENTIONS* **4**, 281-286 (2011). https://doi.org/10.1016/j.jcin.2010.10.015

9 Hashimoto, T. *et al.* Circulating high-mobility group box 1 and cardiovascular mortality in unstable angina and non-ST-segment elevation myocardial infarction. *ATHEROSCLEROSIS* **221**, 490-495 (2012). https://doi.org/10.1016/j.atherosclerosis.2012.01.040

10 Jin, P. *et al.* Elevated preoperative HMGB1 as predictor of myocardial

injury post-percutaneous coronary intervention. *Medicine* **95**, e5149 (2016). https://doi.org/10.1097/MD.0000000000005149

11 Cirillo, P. *et al.* Increased High Mobility Group Box-1 Protein Levels are Associated With Impaired Cardiopulmonary and Echocardiographic Findings After Acute Myocardial Infarction. *JOURNAL OF CARDIAC FAILURE* **15**, 362-367 (2009). https://doi.org/10.1016/j.cardfail.2008.11.010

12 Giallauria, F. *et al.* Autonomic dysfunction is associated with high mobility group box-1 levels in patients after acute myocardial infarction. *ATHEROSCLEROSIS* **208**, 280-284 (2010). https://doi.org/10.1016/j.atherosclerosis.2009.07.025

13 Hu, X. R. *et al.* Increased serum HMGB1 is related to the severity of coronary artery stenosis. *CLINICA CHIMICA ACTA* **406**, 139-142 (2009). https://doi.org/10.1016/j.cca.2009.06.016

14 Huang, J. *et al.* Correlation between high mobility group box 1 protein, tumor necrosis factor-α, and acute coronary syndrome. *BIOMEDICAL RESEARCH-INDIA* **28**, 3798-3803 (2017).

15 Andrassy, M. *et al.* HMGB1 Is Associated with Atherosclerotic Plaque Composition and Burden in Patients with Stable Coronary Artery Disease. *PLOS ONE* **7** (2012). https://doi.org/10.1371/journal.pone.0052081

16 Haghjooy-Javanmard, S., Sadeghi, M., Safavi, S., Gheraati, M. & Dana, N. Prognostic value of the high-mobility group box-1 in young patients with chest pain. *ARYA atherosclerosis* **10**, 154-158 (2014).

17 Wang, J. S. *et al.* Levels of serum high mobility group box 1 were independently associated with cardiovascular risk in patients undergoing coronary angiography. *CLINICA CHIMICA ACTA* **483**, 130-134 (2018). https://doi.org/10.1016/j.cca.2018.04.032

18 Liao, Y. X., Jiang, H. & Hu, X. R. Increased serum IL-17A is associated with HMGB1 in coronary artery disease. *INTERNATIONAL JOURNAL OF CLINICAL AND EXPERIMENTAL MEDICINE* **9**, 11772-11779 (2016).

19 Chen, Q. *et al.* Novel Diagnostic Biomarkers Related to Necroptosis and Immune Infiltration in Coronary Heart Disease. *JOURNAL OF INFLAMMATION RESEARCH* **17**, 4525-4548 (2024). https://doi.org/10.2147/JIR.S457469

20 Stark, K. *et al.* Disulfide HMGB1 derived from platelets coordinates venous thrombosis in mice. *Blood* **128**, 2435-2449 (2016). https://doi.org/10.1182/blood-2016-04-710632

21 Zhao, X. Y. *et al.* High mobility group box 1 derived mainly from platelet

microparticles exacerbates microvascular obstruction in no reflow. *THROMBOSIS RESEARCH* **222**, 49-62 (2023). https://doi.org/10.1016/j.thromres.2022.12.003

22 Rath, D., Geisler, T., Gawaz, M. & Vogel, S. HMGB1 Expression Level in Circulating Platelets is not Significantly Associated with Outcomes in Symptomatic Coronary Artery Disease. *CELLULAR PHYSIOLOGY AND BIOCHEMISTRY* **43**, 1627-1633 (2017). https://doi.org/10.1159/000482026

23 Wahid, A. *et al.* Serum HMGB1 is a biomarker for acute myocardial infarction with or without heart failure. *CTS-CLINICAL AND TRANSLATIONAL SCIENCE* **16**, 2299-2309 (2023). https://doi.org/10.1111/cts.13630

24 Bruton, M. *et al.* Expression of High Mobility Group Protein B1 in Cardiac Tissue of Elderly Patients with Coronary Artery Disease with or without Inflammatory Rheumatic Disease. *GERONTOLOGY* **63**, 337-349 (2017). https://doi.org/10.1159/000471763

25 Lu, C. *et al.* Sex-specific differences in cytokine signaling pathways in circulating monocytes of cardiovascular disease patients. *ATHEROSCLEROSIS* **384** (2023). https://doi.org/10.1016/j.atherosclerosis.2023.04.005

26 Dong, H. F. & Ai, C. The Effect of Amlodipine Combined with Atorvastatin on the Levels of HMGB-1 and YKL-40 in Patients with Coronary Heart Disease and Carotid Atherosclerosis. *INDIAN JOURNAL OF PHARMACEUTICAL SCIENCES* **83**, 174-179 (2021). https://doi.org/10.36468/pharmaceutical-sciences.spl.391

27 Hadi, N. R., Ghazi, A., Amber, K. I., Majeed, S. A. & Al-Aubaidy, H. A. The impact of atorvastatin reload in patients undergoing percutaneous coronary intervention and its correlation with the Toll-like receptors. *Systematic Reviews in Pharmacy* **10**, 53-59 (2019). https://doi.org/10.5530/srp.2019.2.10

28 Desouky, D. A., Nosair, N. A., Sherif, D. E., El-Magd, M. A. & Salama, M. K. Association between circulatory microRNA-218 expression, serum PCSK9 levels, inflammatory markers, and monocyte subsets in coronary artery disease patients: impact of statin therapy. *BENI-SUEF UNIVERSITY JOURNAL OF BASIC AND APPLIED SCIENCES* **13** (2024). https://doi.org/10.1186/s43088-024-00515-8

29 Handayani, W., Suharjono & Yogiarto, M. Analysis of HMGB-1 level before and after providing atorvastatin standard therapy in coronary artery disease patients with type-2 diabetes mellitus compared to without type-

2 diabetes mellitus. *Journal of basic and clinical physiology and pharmacology* **32**, 439-446 (2021). https://doi.org/10.1515/jbcpp-2020-0442
30 Kümmel, A. *et al.* High-Mobility Group Box Protein 1 Is an Independent Prognostic Marker for All-Cause Mortality in Patients With Dilated Cardiomyopathy. *AMERICAN JOURNAL OF CARDIOLOGY* **178**, 119-123 (2022). https://doi.org/doi:10.1016/j.amjcard.2022.05.014
31 Fan, J. J. *et al.* NOD-like receptor protein 3 and high mobility group box-1 are associated with prognosis of patients with congenital heart disease. *JOURNAL OF INTERNATIONAL MEDICAL RESEARCH* **48** (2020). https://doi.org/doi:10.1177/0300060519884500
32 Huang, Y. Y. *et al.* Elevated serum HMGB1 in pulmonary arterial hypertension secondary to congenital heart disease. *VASCULAR PHARMACOLOGY* **85**, 66-72 (2016). https://doi.org/doi:10.1016/j.vph.2016.08.009
33 Caracci, B. *et al.* Circulating High Mobility Group Box-1 Does Not Predict Pulmonary Arterial Hypertension in Children with Congenital Heart Disease: A Prospective Cohort Study. *JOURNAL OF CARDIOTHORACIC AND VASCULAR ANESTHESIA* **38**, 2356-2361 (2024). https://doi.org/doi:10.1053/j.jvca.2024.07.020
34 Kido, T. *et al.* The administration of high-mobility group box 1 fragment prevents deterioration of cardiac performance by enhancement of bone marrow mesenchymal stem cell homing in the delta-sarcoglycan-deficient hamster. *PLOS ONE* **13** (2018). https://doi.org/doi:10.1371/journal.pone.0202838
35 Farini, A. *et al.* PTX3 Predicts Myocardial Damage and Fibrosis in Duchenne Muscular Dystrophy. *FRONTIERS IN PHYSIOLOGY* **11** (2020). https://doi.org/doi:10.3389/fphys.2020.00403
36 Jia, P., Wu, N., Jia, D. & Sun, Y. Downregulation of MALAT1 alleviates saturated fatty acid-induced myocardial inflammatory injury via the miR-26a/HMGB1/TLR4/NF-kappaB axis. *Diabetes, Metabolic Syndrome and Obesity* **12**, 655-665 (2019). https://doi.org/doi:https://dx.doi.org/10.2147/DMSO.S203151
37 Perez, L. M., de Lucas, B., Bernal, A. & Galvez, B. G. Adipokines disrupt cardiac differentiation and cardiomyocyte survival. *INTERNATIONAL JOURNAL OF OBESITY* **44**, 908-919 (2020). https://doi.org/doi:10.1038/s41366-019-0455-4
38 Soultan, K. M. & Carrillo-Sepulveda, M. A. Western diet increases HMGB1

levels and activates cardiac and hepatic Toll-like receptor 4 (TLR4) signaling in male mice. *FASEB Journal* **33**, 505.522 (2019). https://doi.org/doi:https://dx.doi.org/10.1096/fasebj.2019.33.1_supplement.505.22

39 Arrigo, T. *et al.* Cardiac dysfunction in children with essential obesity: preliminary data. *Journal of biological regulators and homeostatic agents* **33**, 79-Nephrology (2019). https://doi.org/doi:

40 Dozio, E. *et al.* Expression of the Receptor for Advanced Glycation End Products in Epicardial Fat: Link with Tissue Thickness and Local Insulin Resistance in Coronary Artery Disease. *JOURNAL OF DIABETES RESEARCH* **2016** (2016). https://doi.org/doi:10.1155/2016/2327341

41 Karuppagounder, V. *et al.* Modulation of Macrophage Polarization and HMGB1-TLR2/TLR4 Cascade Plays a Crucial Role for Cardiac Remodeling in Senescence-Accelerated Prone Mice. *PLOS ONE* **11** (2016). https://doi.org/doi:10.1371/journal.pone.0152922

42 Liu, R. Y., Meng, J. & Lou, D. F. Adiponectin inhibits D-gal-induced cardiomyocyte senescence via AdipoR1/APPL1. *MOLECULAR MEDICINE REPORTS* **24** (2021). https://doi.org/doi:10.3892/mmr.2021.12358

43 Zhang, C. *et al.* High-mobility group box 1 (HMGB1) impaired cardiac excitation-contraction coupling by enhancing the sarcoplasmic reticulum (SR) Ca2+ leak through TLR4-ROS signaling in cardiomyocytes. *Journal of Molecular and Cellular Cardiology* **74**, 260-273 (2014). https://doi.org/doi:https://dx.doi.org/10.1016/j.yjmcc.2014.06.003

44 Liu, W. *et al.* High-mobility group box 1 (HMGB1) downregulates cardiac transient outward potassium current (Ito) through downregulation of Kv4.2 and Kv4.3 channel transcripts and proteins. *Journal of molecular and cellular cardiology* **49**, 438-448 (2010). https://doi.org/doi:https://dx.doi.org/10.1016/j.yjmcc.2010.05.004

45 Tzeng, H. P. *et al.* Negative inotropic effects of high-mobility group box 1 protein in isolated contracting cardiac myocytes. *American Journal of Physiology - Heart and Circulatory Physiology* **294**, H1490-H1496 (2008). https://doi.org/doi:https://dx.doi.org/10.1152/ajpheart.00910.2007

46 Yu, X. M. *et al.* Selective ablation of ligament of Marshall inhibits ventricular arrhythmias during acute myocardial infarction: Possible mechanisms. *JOURNAL OF CARDIOVASCULAR ELECTROPHYSIOLOGY* **30**, 374-382 (2019). https://doi.org/doi:10.1111/jce.13802

47 Pang, L., Yin, J., Shi, Y. G., Wang, Y. & Yan, S. H. High mobility group box-1 in hypothalamic paraventricular nuclei attenuates sympathetic tone in

rats at post-myocardial infarction. *CARDIOLOGY JOURNAL* **26**, 555-563 (2019). https://doi.org/doi:10.5603/CJ.a2018.0117
48 Jiang, F. *et al.* Prediction of herbal medicines based on immune cell infiltration and immune- and ferroptosis-related gene expression levels to treat valvular atrial fibrillation. *FRONTIERS IN GENETICS* **13** (2022). https://doi.org/doi:10.3389/fgene.2022.886860
49 Li, L., Liu, Y., Li, X. & Qiu, C. Insights into the genetic basis of HMGB1 in atrial fibrillation in a Chinese Han population. *Cardiovascular Diagnosis and Therapy* **10**, 388-395 (2020). https://doi.org/doi:https://dx.doi.org/10.21037/cdt.2019.12.07
50 Qu, C. *et al.* High mobility group box 1 gene polymorphism is associated with the risk of postoperative atrial fibrillation after coronary artery bypass surgery. *Journal of cardiothoracic surgery* **10**, 88 (2015). https://doi.org/doi:https://dx.doi.org/10.1186/s13019-015-0301-2
51 Wu, Y. *et al.* Increased serum HMGB1 is related to oxidative stress in patients with atrial fibrillation. *The Journal of international medical research* **41**, 1796-1802 (2013). https://doi.org/doi:https://dx.doi.org/10.1177/0300060513503917
52 Li, X. *et al.* Predictive value of HMGB1 for atrial fibrillation recurrence after cryoballoon ablation in paroxysmal atrial fibrillation patients. *Clinical cardiology* **45**, 1229-1235 (2022). https://doi.org/doi:https://dx.doi.org/10.1002/clc.23904
53 Xu, Q. *et al.* High mobility group box 1 was associated with thrombosis in patients with atrial fibrillation. *Medicine* **97**, e0132 (2018). https://doi.org/doi:https://dx.doi.org/10.1097/MD.0000000000010132
54 Bai, J. *et al.* The Effect of PD-1 Inhibitor Combined with Irradiation on HMGB1-Associated Inflammatory Cytokines and Myocardial Injury. *Journal of Inflammation Research* **15**, 6357-6371 (2022). https://doi.org/doi:https://dx.doi.org/10.2147/JIR.S384279
55 Huang, Y. F., Cheng, M. H., Wang, X. Y., Dong, H. L. & Gao, J. Dang Gui Bu Xue Tang, a conventional Chinese herb decoction, ameliorates radiation-induced heart disease <i>via</i> Nrf2/HMGB1 pathway. *FRONTIERS IN PHARMACOLOGY* **13** (2023). https://doi.org/doi:10.3389/fphar.2022.1086206
56 Goto, K. *et al.* Berberine Improves Cancer-Derived Myocardial Impairment in Experimental Cachexia Models by Targeting High-Mobility Group Box-1. *International Journal of Molecular Sciences* **25**, 4735 (2024). https://doi.org/doi:https://dx.doi.org/10.3390/ijms25094735
